# Novel detection of post-translational modifications in human monocyte-derived dendritic cells after chronic alcohol exposure: Role of inflammation regulator H4K12ac

**DOI:** 10.1038/s41598-017-11172-6

**Published:** 2017-09-11

**Authors:** Tiyash Parira, Gloria Figueroa, Alejandra Laverde, Gianna Casteleiro, Mario E. Gomez Hernandez, Francisco Fernandez-Lima, Marisela Agudelo

**Affiliations:** 1 Department of Immunology, Herbert Wertheim College of Medicine, Florida International University, Miami, FL 33199 United States; 2 Advanced Mass Spectrometry Facility, Department of Chemistry and Biochemistry, Florida International University, Miami, FL 33199 United States

## Abstract

Previous reports on epigenetic mechanisms involved in alcohol abuse have focus on hepatic and neuronal regions, leaving the immune system and specifically monocyte-derived dendritic cells (MDDCs) understudied. Our lab has previously shown histone deacetylases are modulated in cells derived from alcohol users and after *in vitro* acute alcohol treatment of human MDDCs. In the current study, we developed a novel screening tool using matrix assisted laser desorption ionization-fourier transform-ion cyclotron resonance mass spectrometry (MALDI-FT-ICR MS) and single cell imaging flow cytometry to detect post-translational modifications (PTMs) in human MDDCs due to chronic alcohol exposure. Our results demonstrate, for the first time, *in vitro* chronic alcohol exposure of MDDCs modulates H3 and H4 and induces a significant increase in acetylation at H4K12 (H4K12ac). Moreover, the Tip60/HAT inhibitor, NU9056, was able to block EtOH-induced H4K12ac, enhancing the effect of EtOH on IL-15, RANTES, TGF-β1, and TNF-α cytokines while restoring MCP-2 levels, suggesting that H4K12ac may be playing a major role during inflammation and may serve as an inflammation regulator or a cellular stress response mechanism under chronic alcohol conditions.

## Introduction

The notion that an epigenetic modification can be described as a heritable change in genetic composition that is not directly encoded in the DNA^1^ has fascinated researchers over time. In the context of addiction, epigenetic modifications have been implicated in alcohol abuse^2–5^. Chronic alcohol use, one of the leading causes of morbidity, may result in addiction, which is intrinsically related to brain behavior and neuronal remodeling^6^. Recently, researchers have begun to investigate the epigenetic effects of alcohol abuse; however, most studies have been conducted in the hepatic^2^ or neuronal regions^7,8^ as recently reviewed by us^9^. Besides the vast amount of literature regarding the effects of alcohol abuse on the liver and CNS, alcohol is also known to affect the human immune system by modulating both the innate and adaptive components of the immune system^10^.

Despite previous research in the alcoholism and epigenetics fields, little is known about the epigenetic effects of chronic alcohol exposure in the human immune system. Therefore, the current study aims to reveal the epigenetic effects of chronic alcohol treatment in monocyte-derived dendritic cells (MDDCs), specifically the ability of chronic alcohol to induce histone modifications and subsequent functional effects. The epigenetic effects of chronic alcohol were assessed through the analysis of H3 and H4 modifications with a focus on histone (H)4 lysine (K)12 acetylation(ac). Results from this study provide novel insights into the epigenetic effects of chronic alcohol in the periphery and reveal H4K12ac as a novel epigenetic post-translational modification marker for chronic alcohol.

## Results

### Chronic treatment of MDDCs with 0.2% alcohol (EtOH) leads to increases in histone H3 and H4 quantity

**Figure 1:**
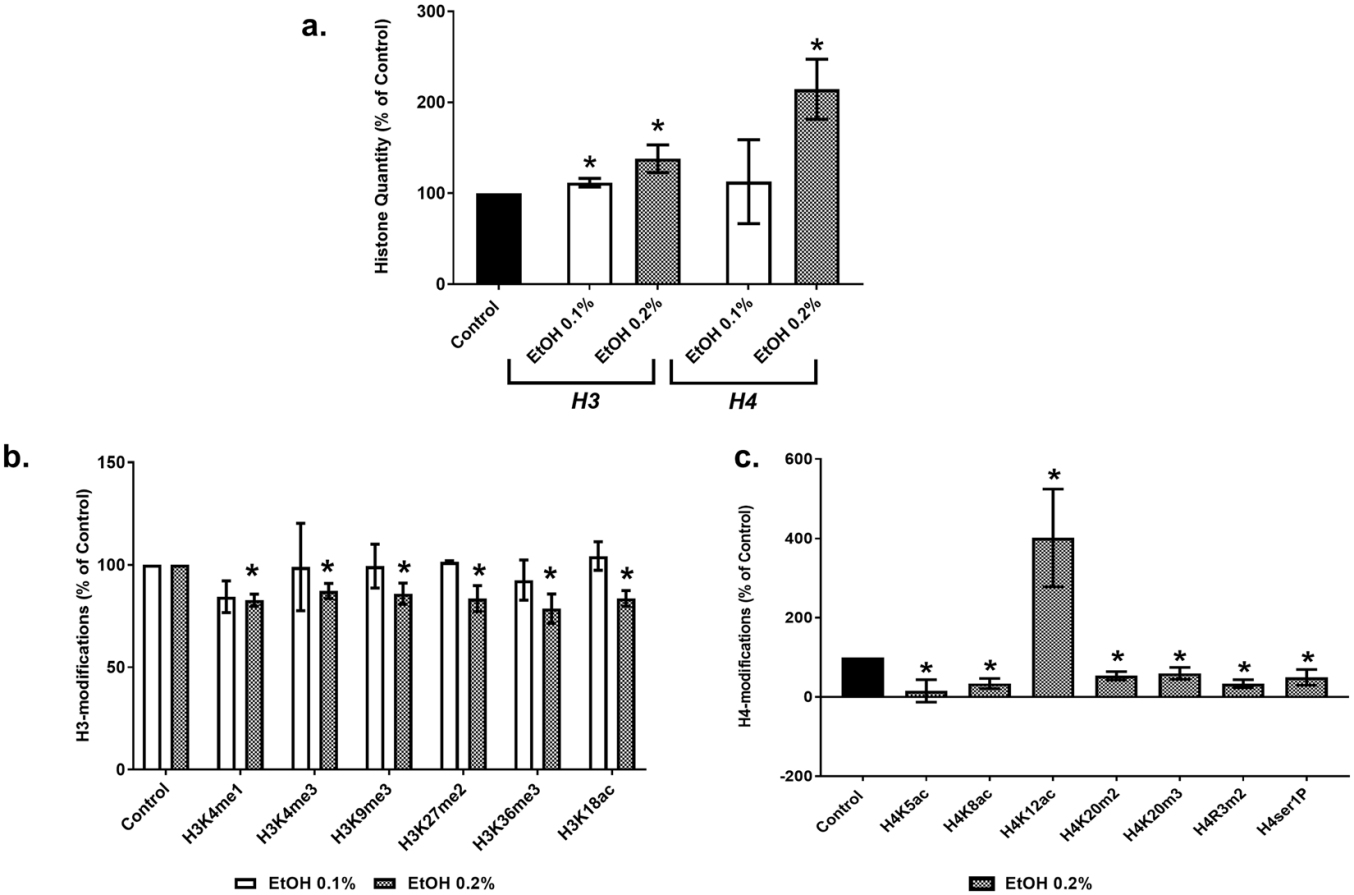
Chronic treatment of MDDCs with alcohol (EtOH) leads to increased histone H3 and H4 quantity followed by modulation in H3 and H4 modifications. After 5–7 days of differentiation, MDDCs were treated with 0.1%, and 0.2% alcohol for 5 days. Panel a: Histone H3 and H4 quantity was analyzed by ELISA-based EpiQuik quantification kits independent of its modified state. The values are displayed as percent of control ± SEM from at least five independent experiments. Graph indicates H3 at EtOH 0.1% (111.6 ± 4.858, p = 0.033) and 0.2% (138.1 ± 15.18, p = 0.027), H4 at EtOH 0.2% (214.5 ± 33.02, p = 0.0017). Panel b: Histone extracts from MDDCs chronically treated with EtOH 0.1 and 0.2% were analyzed for 21 H3 post-translational modification states. Results show levels of significantly altered histone modifications for 0.1 and 0.2% EtOH, presented as percentage over untreated control ± SEM from two (EtOH 0.1%) and three (EtOH 0.2%) independent experiments. Chronic treatment with 0.2% EtOH in MDDCs showed significant downregulation at the sites of H3K4me1 (82.78% ± 2.95, p = 0.004), H3K4me3 (87.25% ± 3.67, p = 0.025), H3K9me3 (85.97% ± 5.17, p = 0.053), H3K27me2 (83.54% ± 6.30, p = 0.059), H3K36me3 (78.63% ± 7.17, p = 0.041) and H3K18ac (83.62% ± 3.80, p = 0.013) in comparison to untreated control. Panel (c): Histone extracts from MDDCs chronically treated with EtOH 0.2% were analyzed for 10 H4 post-translational modification states. Results show levels of significantly altered histone modifications for EtOH 0.2% presented as percentage over untreated control ± SEM from 7 (EtOH 0.2%) independent experiments. Chronic treatment of the EtOH 0.2% in MDDCs showed significant downregulation at H4K5ac (15.44% ± 28.31, p = 0.011), H4K8ac (33.69% ± 12.89, p = 0.0002), H4K20m2 (53.63% ± 10.55, p = 0.0009), H4K20m3 (59.63% ± 10.55, p = 0.019), H4R3m2 (33.45 ± 10.27, p = 0.0003) and H4ser1P (49.36 ± 19.86, p = 0.025) and upregulation at H4K12ac (401.64% ± 123.40, p = 0.031), in comparison to untreated control. Statistical differences were calculated using student’s t-test when individually compared to untreated control and significant differences are indicated with*.

To measure total levels of histones H3 and H4, quantifications were performed for MDDCs treated with 0.1 and 0.2% EtOH for 5 days, using an enzyme linked immunosorbent assay (ELISA) EpiQuik quantification technique. As shown in Fig. 1 panel a, total H3 was significantly upregulated after 0.1% EtOH (111.6 ± 4.858, p = 0.033) and 0.2% EtOH (138.1 ± 15.18, p = 0.027) while total H4 was significantly upregulated only at 0.2% EtOH (214.5 ± 33.02, p = 0.0017) when compared to untreated control. Although there was an overall unbalanced increase of H3 and H4 quantity after EtOH exposure, when statistical analysis was performed comparing the quantity of H3 versus H4 among the 0.1 and 0.2% EtOH-treated groups, there was no significant differences. Supplementary Figure S1 shows representative standard curves used for H3 and H4 quantification.

### Chronic treatment of MDDCs with 0.2% EtOH reveals significant downregulation of H3 post-translational modifications

As shown in Fig. 1 panel b, chronic treatment of MDDCs with 0.2% EtOH downregulated H3K4me1 (82.78% ± 2.95, p = 0.004), H3K4me3 (87.25% ± 3.67, p = 0.025), H3K9me3 (85.97% ± 5.17, p = 0.053), H3K27me2 (83.54% ± 6.30, p = 0.059), H3K36me3 (78.63% ± 7.17, p = 0.041) and H3K18ac (83.62% ± 3.80, p = 0.013) in comparison to untreated MDDCs. However, treatment with 0.1% EtOH did not significantly modulate H3 modification sites. Although no significant dose-dependent downregulation was found through statistical analyses, treatment with 0.2% EtOH decreased post-translational modification status with respect to treatment with 0.1% EtOH. Supplementary Table T1 includes the mean % of control, SEM, and p values for 21 H3 modifications after 0.1 and 0.2% EtOH treatments.

### Chronic treatment of MDDCs with 0.2% EtOH modulates H4 sites and significantly up-regulates H4K12ac

**Figure 2:**
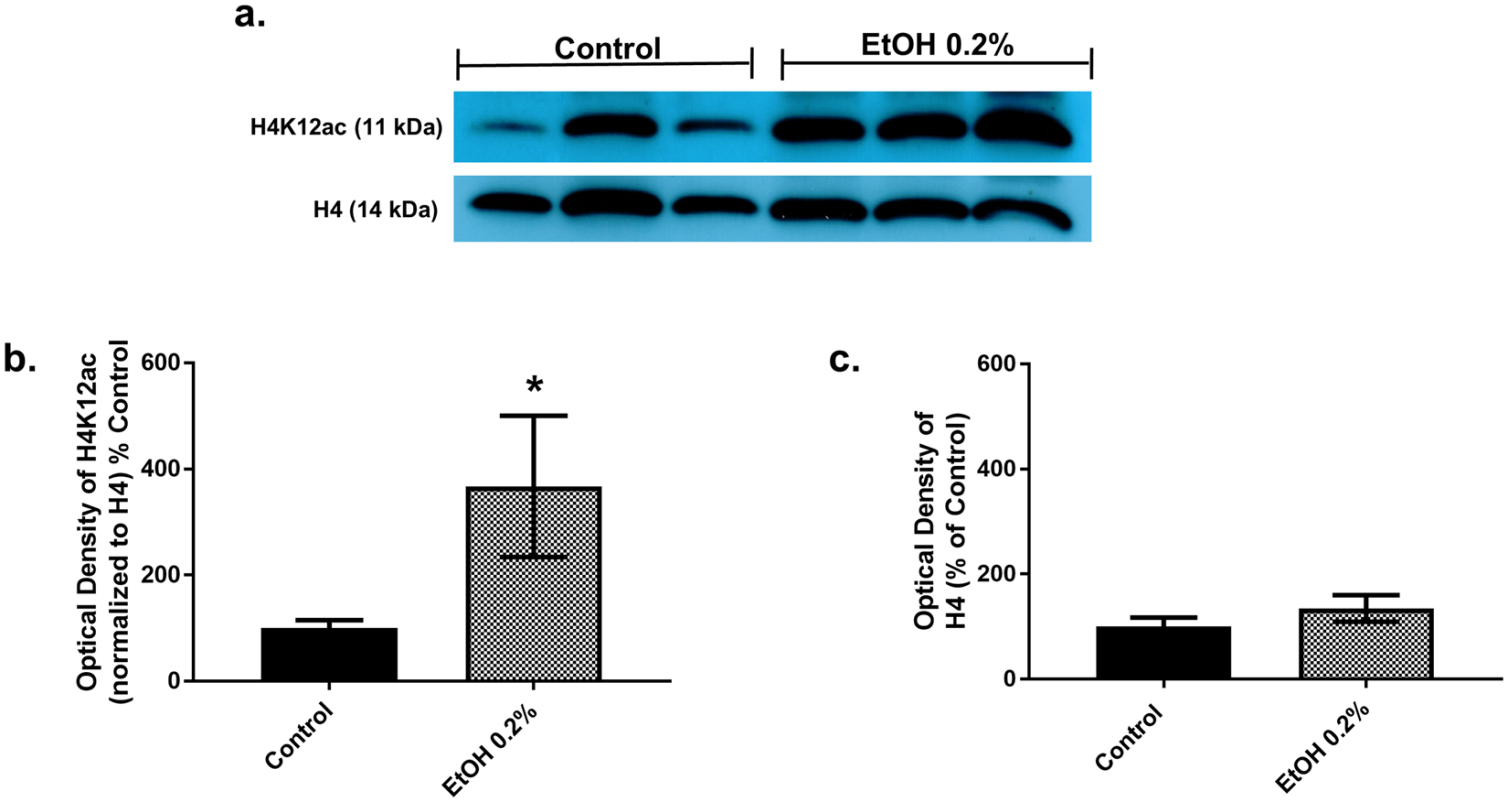
Chronic treatment of MDDCs with 0.2% EtOH increases H4K12ac as measured by Immunoblotting. Western blot was carried out for H4K12ac and H4 in histone extracts from untreated control or 0.2% EtOH treated MDDCs. Panel a- shows representative blot highlighting the protein of interest (full length blots are included in the Supplementary Figure S4), where first 3 lanes have histone extracts from three biological replicates of untreated MDDCs and next 3 lanes contain histone extracts from three biological replicates of MDDCs chronically treated with 0.2% EtOH. The H4K12ac band appears at 11 kDa while the H4 band appears at 14 kDa. Panel b - optical density of H4K12ac normalized to H4 when analyzed by ImageJ and represented as % control. MDDCs chronically treated with 0.2% EtOH (367.3 %± 132.9, p = 0.05) showed a higher expression of H4K12ac in comparison to untreated MDDCs (100 %± 15.05). Panel c - graphical representation of the optical density (OD) values of the H4 band. 0.2% EtOH treated MDDCs (134.6% ± 25.15) contain increased amount of H4 compared to control (100% ± 17.19). Western blot was carried out with histone extracts for control and 0.2% EtOH treated MDDCs from at least 13 different buffy coats. Statistical differences were calculated using t-test when compared to untreated control.

As shown in Fig. 1 panel c, chronic EtOH treatment of MDDCs with 0.2% EtOH, significantly downregulated H4K5ac (15.44% ± 28.31, p = 0.011), H4K8ac (33.69% ± 12.89, p = 0.0002), H4K20m2 (53.63% ± 10.55, p = 0.0009), H4K20m3 (59.63% ± 10.55, p = 0.019), H4R3m2 (33.45% ± 10.27, p = 0.0003) and H4ser1P (49.36% ± 19.86, p = 0.025) while significantly upregulating H4K12ac (401.64% ± 123.40, p = 0.031) in comparison to untreated MDDCs. Supplementary Table T2 displays the mean % of control, SEM, and *p* values for 10 H4 modifications after 0.2% EtOH treatment. To further confirm the upregulation at H4K12ac, we performed immunoblotting. Figure 2 panel a shows a representative blot depicting H4K12ac expression in histone extracts from MDDCs untreated and chronically treated with 0.2% EtOH. Full blots are included in the Supplementary Figure S4. Figure 2 panel c shows a graphical representation of the optical density (OD) values of the H4 band as analyzed by ImageJ and represented as percent of control. 0.2% EtOH treated MDDCs (134.6% ± 25.15) contain increased (not significant) amount of H4 compared to control (100% ± 17.19). Figure 2 panel b shows OD of H4K12ac analyzed by ImageJ, normalized to H4 and represented as percent of control as previously reported^11–13^. MDDCs chronically treated with 0.2% EtOH (367.3 %± 132.9, p = 0.05) show a significantly higher expression of H4K12ac in comparison to untreated MDDCs (100% ± 15.05).

### MALDI-FT-ICR-MS confirms acetylation peaks are enriched in 0.2% EtOH treated MDDCs compared to untreated MDDCs

**Figure 3:**
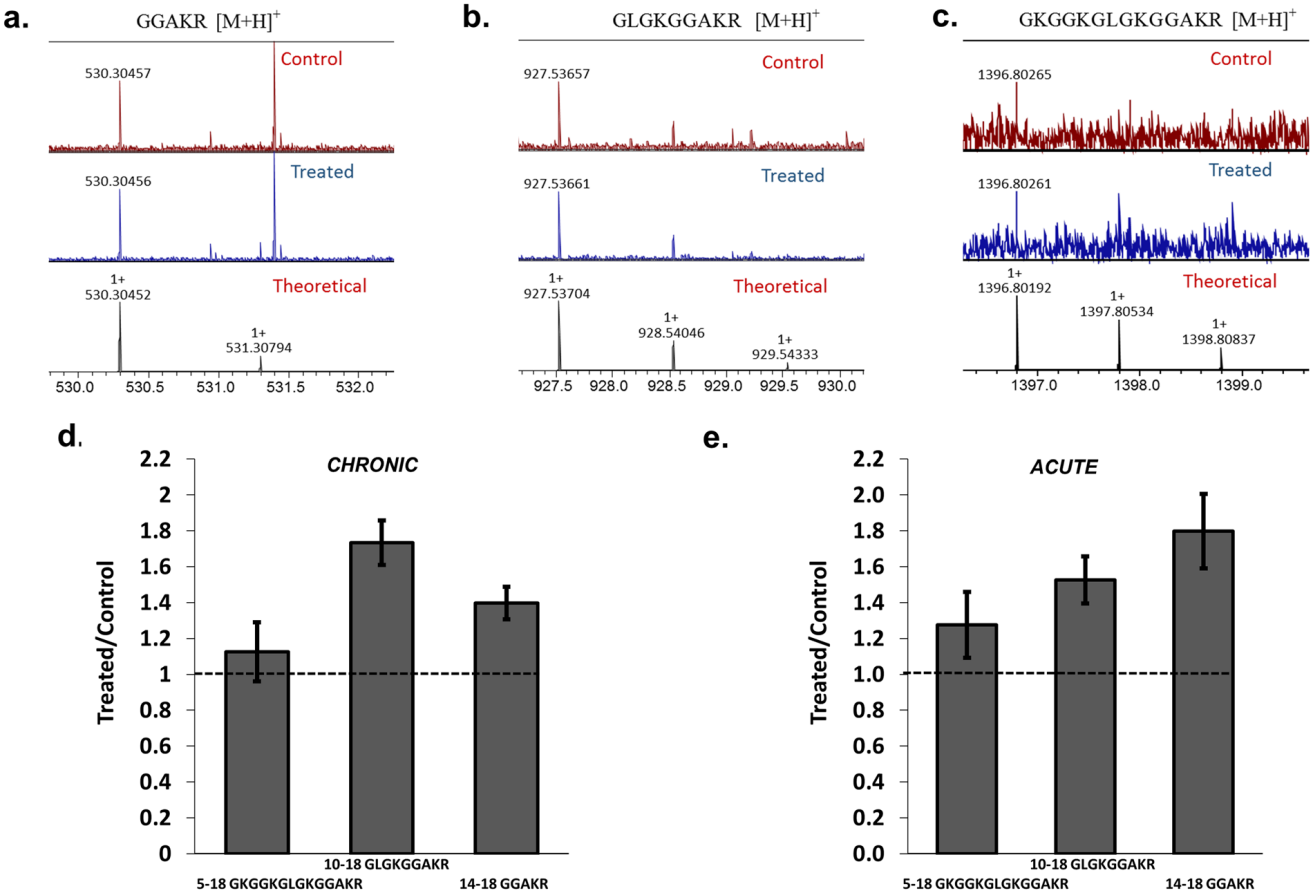
MALDI-FT-ICR-MS confirms acetylation peaks are enriched in 0.2% EtOH treated MDDCs compared to untreated MDDCs. Typical representative MALDI-FT-ICR MS spectra of the 14–18 GGAKR (Panel a), 10–18 GLGKGGAKR (Panel b) and 5–18 GKGGKGLGKGGAKR (Panel c) in the control and 0.2% EtOH treated MDDC samples (theoretical isotopic pattern is shown for all cases). There is higher abundance of the acetylated peptides in the chronic 0.2% EtOH-treated MDDCs relative to the control MDDCs (Panel d). Relative abundance of acetylated peptides in MDDCs acutely treated with 0.2% EtOH compared to untreated control is shown in panel e. Relative abundances were normalized using internal, non-acetylated common peptides (Supplementary Figures S2 and S3).

To further verify the increased acetylation in MDDCs chronically treated with 0.2% EtOH, we carried out SDS-PAGE separation of histone proteins, in-gel tryptic digestion of histone H4 followed by MALDI-FT-ICR MS for untreated and 0.2% EtOH-treated MDDCs simultaneously. Three peptides containing acetylation were observed in untreated MDDCs and 0.2% EtOH (see Table 1); other detected non-acetylated tryptic digest peptides are summarized in Supplementary Table T3. As shown in Fig. 3, the control and the 0.2% EtOH-treated MDDCs H4 have K16 acetylated as indicated by the *m/z* 530.3045 (panel a) signal with higher relative abundances on the acetylated peptide in the treated MDDCs compared to acetylated peptide in the control MDDCs (1.12 + /− 0.16 times) (panel d). Furthermore, a peptide signal was detected containing acetylation at K12 and K16 at m/z 927.5365 (panel b) for the control and treated MDDCs, respectively, with higher relative abundances in the treated compared to the control MDDCs (1.73 + /− 0.12 times) (panel d). A peptide signal was detected at *m/z* 1396.8026 (panel c) for the control and treated MDDCs corresponding to the acetylation of K5, K12 and K16; in this case, a higher relative abundance was also observed for the treated MDDCs compared to the control MDDCs (1.39 + /− 0.09 times) (panel d). These observations suggest higher levels of acetylation in MDDCs treated chronically with 0.2% EtOH compared to the untreated MDDCs. In order to address how EtOH exposure progressively changes histone modifications, MALDI-FT-ICR MS was also carried out after MDDCs were treated acutely (24 hours) with 0.2% EtOH. Results are shown in panel e, and confirms that the same peptide signals containing acetylations K5, K12 and K16 were detected (see Table 2) acutely and chronically. However, after acute treatments the relative abundance of peptide for acetylated K12 and K16 at *m/z* 927.5373 for treated was 1.53 + /− 0.13 times higher compared to control; however, it was lower when compared to chronic alcohol-treated studies. Furthermore, relative abundance of peptide for acetylated K16 at *m/z* 530.3046 for acutely treated samples was 1.80 + /−0.21 times higher compared to control. Therefore, acetylation at K12 gradually increases from acute to chronic treatment confirming that EtOH exposure progressively changes histone modifications over time. Supplementary Figure S2 panel a depicts the relative abundance of the acetylated peaks in the control and chronically treated MDDCs normalized to common non-acetylated peptide signals (e.g., *m/z* 1180.6216). In addition, Supplementary Figure S2 panel b shows the MALDI-FT-ICR MS spectra of the control and chronically treated MDDCs. Supplementary Figure S3 panel a and b depict the same for acutely treated MDDCs. The observed peptides and acetylation sites are consistent with previously reported peptide mass mapping of acetylated isoforms of H4^14^.

**Table 1:** Acetylated peptides detected by MALDI-FT-ICR MS for control and chronically treated MDDCs.

Sample	Measured m/z	Theoretical [M + H]^+^	error (ppm)	Sequence	Range
Control	1396.80265	1396.80192	0.52	G**K**GG**K**GLG**K**GGA**K**R	5–18
	927.53657	927.53704	−0.51	GLG**K**GGA**K**R	10–18
	530.30457	530.30452	0.09	GGA**K**R	14–18
Treated 0.2% EtOH Chronic	1396.80261	1396.80192	0.49	G**K**GG**K**GLG**K**GGA**K**R	5–18
	927.53661	927.53704	−0.47	GLG**K**GGA**K**R	10–18
	530.30456	530.30452	0.08	GGA**K**R	14–18

**Table 2:** Acetylated peptides detected by MALDI-FT-ICR MS for control and acutely treated MDDCs.

Sample	Measured m/z	Theoretical [M+H]^+^	error (ppm)	Sequence	Range
Control	1396.80167	1396.80192	−0.18	G**K**GG**K**GLG**K**GGA**K**R	5–18
	927.53689	927.53704	−0.16	GLG**K**GGA**K**R	10–18
	530.30459	530.30452	0.13	GGA**K**R	14–18
Treated 0.2% EtOH Acute	1396.8015	1396.80192	−0.3	G**K**GG**K**GLG**K**GGA**K**R	5–18
	927.53732	927.53704	0.29	GLG**K**GGA**K**R	10–18
	530.30462	530.30452	0.19	GGA**K**R	14–18

### Upregulation of H4K12ac due to chronic treatment with 0.2% EtOH is restored to basal levels by Tip60/HAT inhibitor, NU9056

**Figure 4:**
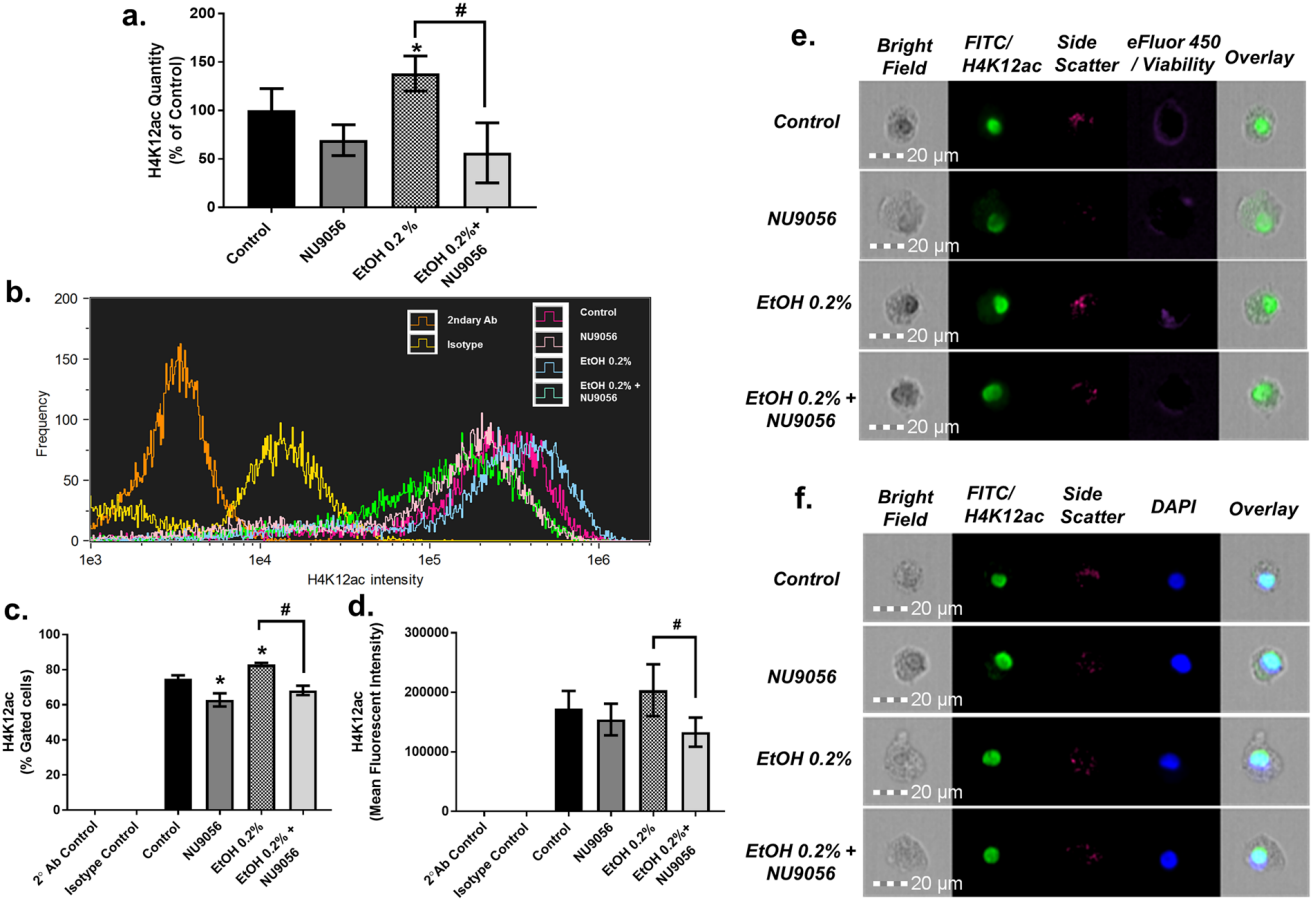
Upregulation of H4K12ac due to chronic treatment with 0.2% EtOH is restored to basal levels by Tip60/HAT inhibitor, NU9056. Post differentiation, MDDCs were treated with NU9056 50 nM, EtOH 0.2% or both for 5 days. Panel a shows H4K12ac quantity from histone extracts as percent (%) of control ± SEM from at least three independent experiments. Statistical differences were calculated using student’s t-test compared to untreated control (100 ± 22.65) and indicated for H4K12ac at NU 50 nM (69.35 ± 15.96), EtOH 0.2% (138.2 ± 18.09, p = 0.05), and EtOH 0.2% + NU 50 nM (56.27 ± 31, p = 0.04). Interactions between groups were tested by ANOVA which gave a p = 0.008. Intra-nuclear staining for H4K12ac and single cell imaging flow cytometry were also performed and it was carried out at-least three times in triplicates. 10,000 events were acquired from live population (eFLUOR450 dye) per sample. Controls with secondary (2°) antibody (Ab) only and isotype staining were also analyzed. Panel b shows a representative overlay of histogram of intensity of channel 2 (FITC) for each treatment shown in legend on the histogram. Panel c shows the percentage of FITC positive (H4K12ac expressing cells). 2° Ab control showed (0.02% ± 0.009) H4K12ac positive population. Isotype control showed (0.34% ± 0.001) positive for H4K12ac. NU 9056 (50 nM) (62.7% ± 3.5, p = 0.004) reduced H4K12ac positive cells compared to control (74.93% ± 1.73) while 0.2% EtOH increased H4K12ac positive cells (82.9% ± 0.84, p = 0.0008) compared to control. 0.2% EtOH and NU9056 (50 nM) reduced H4K12ac positive cells (68.21% ± 2.57, p = 0.0005) compared to cells treated with 0.2% EtOH alone. ANOVA gave a significant value of p < 0.0001. Panel d shows MFI of H4K12ac population from each treatment after background subtraction. 0.2% EtOH + NU9056 50 nM (133163 ± 23359, p = 0.0002) show significantly reduced MFI compared to 0.2% EtOH (203686 ± 41517) treated MDDCs only. Panel e shows representative single cell images from experiments where live cells were acquired based on staining with fixable viability dye eFLUOR 450. Panel f shows representative single cell images confirming nuclear staining with H4K12ac antibody based on DAPI co localization with H4K12ac.

A significantly upregulated post-translational acetylation event points towards increased activity or functionality of histone acetyl transferases (HATs)^15^. Amongst them, Tip 60, which is known to acetylate H4^16^, has recently been shown to be responsible for acetylating H4K12^17,18^. To further analyze the functional role of H4K12ac in MDDCs under chronic alcohol stress, NU9056, a recently identified Tip60 inhibitor^19^, was used to block EtOH-induced acetylation at H4K12. As measured by ELISA (Fig. 4, panel a), chronic exposure of MDDCs with 50 nM NU9056 (69.35 ± 15.96) (Supplementary Figure S5 shows NU9056 toxicity) reduces H4K12ac lower than basal levels compared to control (100 ± 22.65) while 0.2% EtOH treatment (138.2 ± 18.09, p = 0.05) significantly increases H4K12ac compared to control. Also, when the cells were co-treated with 0.2% EtOH and NU9056 (50 nM) (56.27 ± 31, p = 0.04), there was a significant reduction on H4K12ac compared to 0.2% EtOH-treated cells, confirming the ability of NU9056 to restore the effects of EtOH on H4K12ac. To further validate the ability of NU9056 to inhibit the effect of EtOH on H4K12ac, intra-nuclear staining to detect H4K12ac followed by single cell imaging flow cytometry was carried out. Figure 4, panel b shows a representative colored histogram overlay of intensity of channel 2 (H4K12ac-FITC). For isotype control (yellow), secondary antibody only (orange), control (red), NU9056 50 nM (pink), 0.2% EtOH (blue) and combination of 0.2% EtOH and NU9056 50 nM (green) treated MDDCs. The histogram overlay represents the shift in intensity of H4K12ac positive cells showing a decrease or leftward shift for NU9056 (50 nM), increase or rightward shift for 0.2% EtOH compared to control and a decrease again for combination of 0.2% EtOH and NU9056 (50 nM) in comparison to 0.2% EtOH-treated MDDCs. Panel c shows NU9056 (50 nM) (62.78% ± 3.53, p = 0.004) significantly reduced the percentage of H4K12ac positive cells compared to control (74.93% ± 1.73) while 0.2% EtOH increased the percentage of H4K12ac positive cells (82.95% ± 0.84, p = 0.0008) compared to control. When the cells were treated with a combination of 0.2% EtOH and NU9056 (50 nM), there was a significant reduction on H4K12ac positive cells (68.21% ± 2.57, p = 0.0005) compared to cells treated with 0.2% EtOH alone. Panel d shows mean fluorescent intensity (MFI) of H4K12ac-FITC positive cells. MDDCs treated with NU9056 50 nM (154451 ± 25304) shows reduced MFI compared to untreated MDDCs (172842 ± 28155). MDDCs chronically treated with 0.2% EtOH (203686 ± 41517) show increased MFI compared to control while MDDCs treated with 0.2% EtOH and NU9056 50 nM (133163 ± 23359, p = 0.0002) show significantly reduced MFI compared to 0.2% EtOH treated MDDCs. The results from panel d confirm that NU9056 50 nM in combination with 0.2% EtOH reduce alcohol induced H4K12ac expression in MDDCs. Representative single cell images are shown in panel e. To confirm nuclear co-localization of H4K12ac, treated or untreated MDDCs were labelled with H4K12ac using intra-nuclear staining protocol and followed by staining with 4′,6-Diamidino-2-Phenylindole, Dihydrochloride (DAPI). Images were acquired and panel f shows representative single cell images with last channel showing an overlay of bright field, FITC and DAPI, confirming the nuclear co-localization of H4K12ac. Although most of the staining is localized on the nucleus, there is some presence of cytoplasmic staining for H4K12ac. This could be due to non-specific binding of the antibodies^20,21^. To account for it, staining with isotype and secondary antibody controls was carried out and as evident in Fig. 4 panels b–c they showed very low signal, 0.3% and 0.02%, respectively. Another explanation for the cytoplasmic staining is the presence of extra-nuclear histones due to apoptosis as previously reported in activated human lymphoblasts as an early event in apoptosis^22,23^.

### Inhibition of H4K12ac with NU9056 modulates cytokine and chemokine production

**Figure 5:**
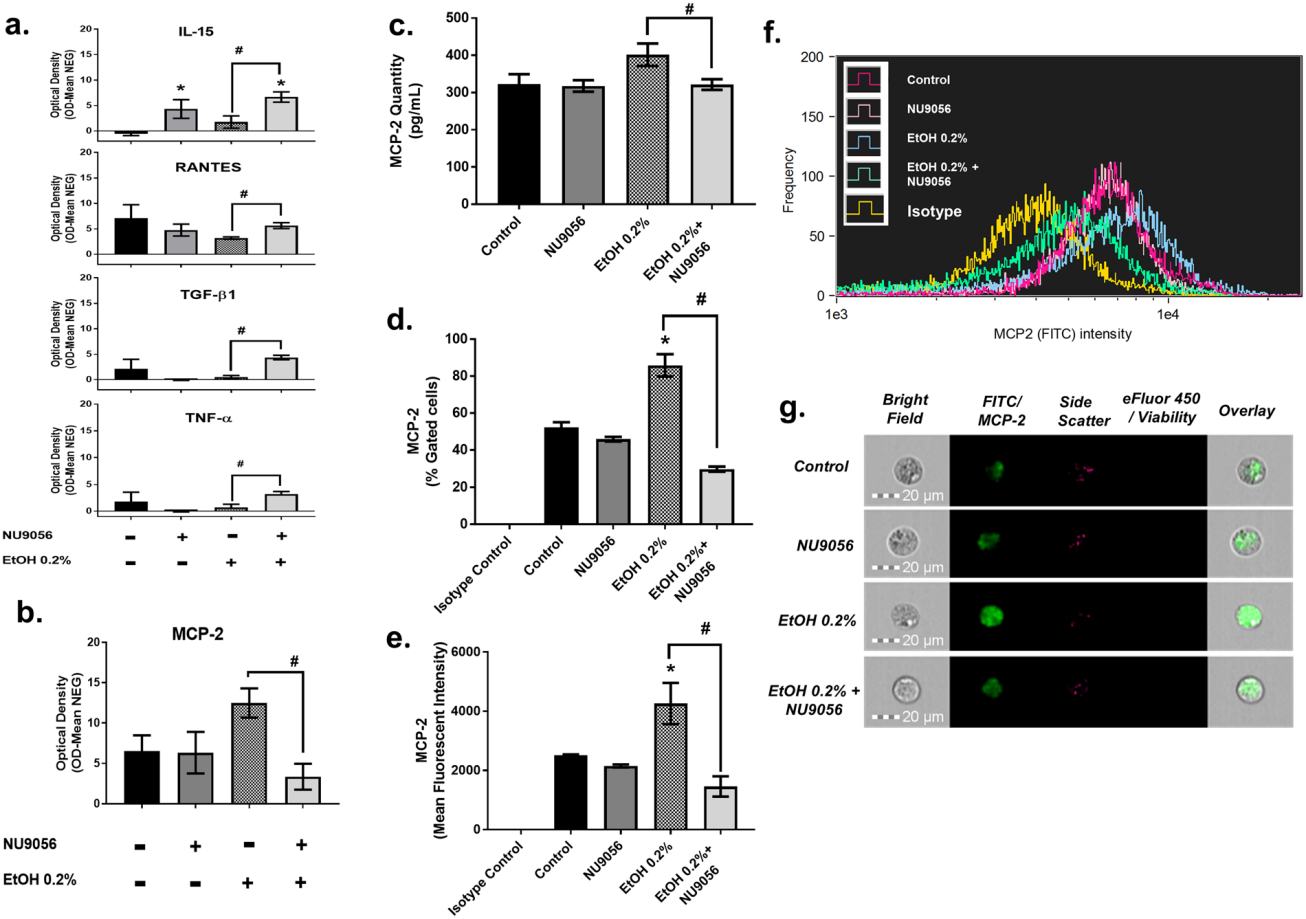
Inhibition of H4K12ac with NU9056 modulates cytokine and chemokine production. After 5–7 days of differentiation, MDDCs were treated with NU9056 50 nM, 0.2% EtOH or both for 5 days. Supernatants collected were analyzed for 48 inflammatory cytokines and chemokines. Data is from 3 blots for untreated control, 2 blots each for treatment with 0.2% EtOH alone, treatment with NU9056 alone, and treatment with 0.2% EtOH plus NU9056. Cytokines chosen to be presented in the graph were selected on the basis of fold change (2 folds or more) compared to control. Each cytokine is detected in duplicates within each blot. Statistical differences were calculated using student’s t-test when individually compared to untreated control and indicated with a * (compared to control) and # (between treatments) for significant p value. Two-way ANOVA was carried out (p = 0.027). Panel a shows optical density values for cytokines IL-15, RANTES, TGF-β1 and TNF-α. Panel b shows optical density values for MCP2. In panel c, MCP-2 ELISA was carried out from cell culture medium at least three times in duplicates. Values detected in pg/mL are as follows, untreated control (322.4 ± 25.04), NU9056 50 nM (317.5 ± 14.3), 0.2% EtOH (401.7 ± 28.2) and 0.2% EtOH + NU9056 50 nM (321.6 ± 13.5, p = 0.03). Panels d–g show intracellular staining to measure MCP-2 levels in untreated or treated MDDCs. Data represented is from at least 3 experiments. Panel d shows % gated MCP-2 positive cells for untreated control (52.3% ± 2.3), NU9056 50 nM (45.8% ± 1.07), 0.2% EtOH (85.8% ± 5.2, p = 0.002) and 0.2% EtOH + NU9056 50 nM (29.6% ± 1.18, p = 0.0001). Panel e shows MFI values for MCP-2-FITC for untreated control (2510 ± 29), NU9056 50 nM (2151 ± 43), 0.2% EtOH (4260 ± 603, p = 0.04) and 0.2% EtOH + NU9056 50 nM (1459 ± 297, p = 0.01). Panel f shows an overlay histogram of MCP-2-FITC for all the samples as shown in legend. Panel g shows representative single cell images.

**Figure 6:**
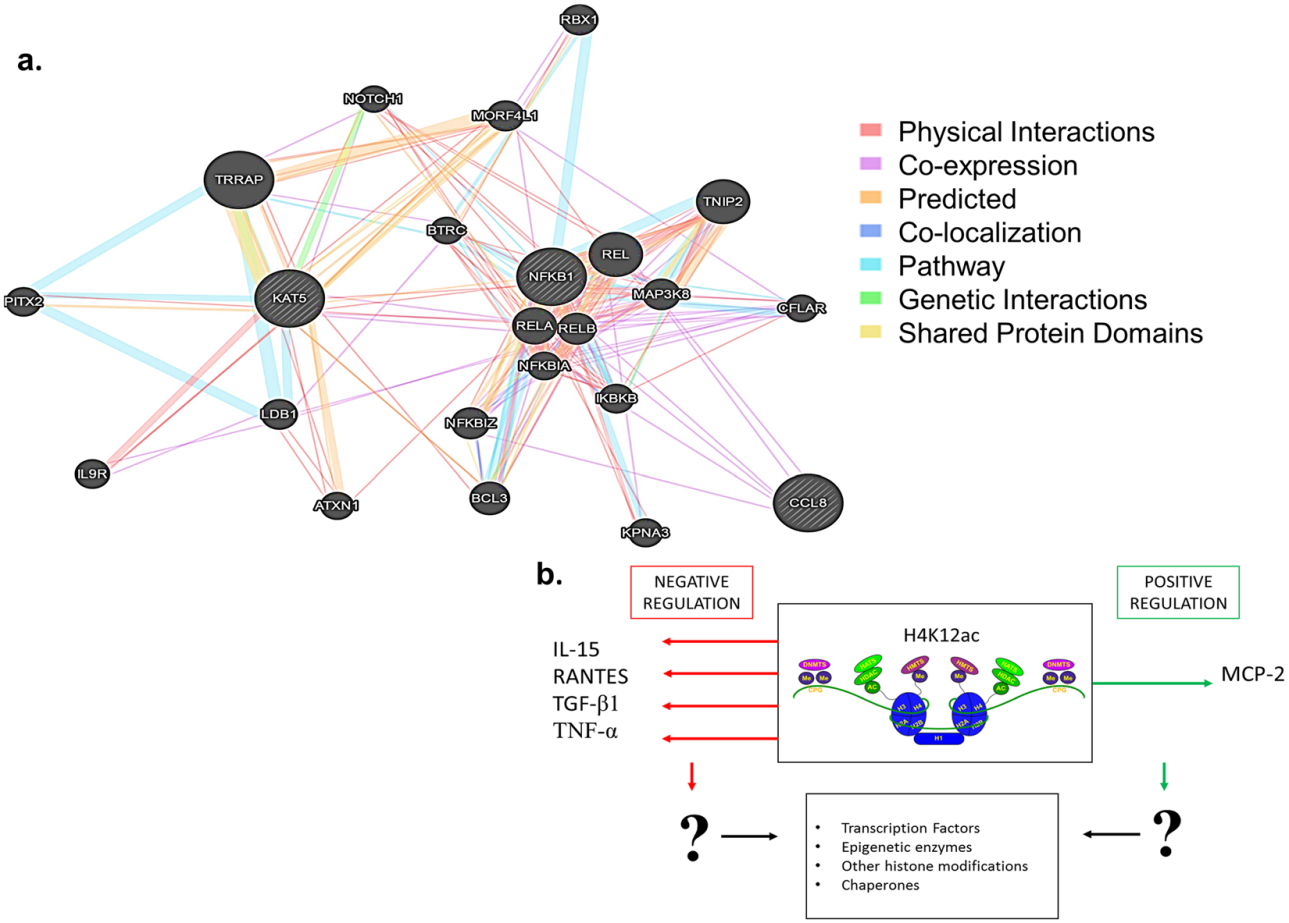
Summary of immunomodulatory role of H4K12ac. Panel a depicts the *in silico* analysis of genes KAT5, NFKB1 and CCL8 revealing several gene interactions using GeneMANIA. Panel b provides a schematic of the cytokine modulation partially adapted from our review^9^. H4K12ac negatively regulates IL-15, RANTES, TGF-β1 and TNF-α while positively regulating MCP-2. However, additional studies are necessary to confirm the immunoregulatory role of H4K12ac and to elucidate the molecular mechanisms behind this epigenetic immunomodulation as recently reviewed by us^9^.

Alcohol has been known to alter cytokine and chemokine secretion by immune cells^24^. Our lab has also previously reported modulated cytokine secretion profile in alcohol users^25^ and after *in-vitro* acute alcohol treatment in MDDCs^26^. In order to study the functional relevance of upregulation of H4K12ac due to chronic alcohol exposure in MDDCs, we looked at secretion of inflammatory cytokine and chemokine profiles under the treatment of NU9056 alone, chronic EtOH treatment, and combination of chronic EtOH and NU9056. From the cytokines that showed modulation after H4K12ac inhibition (Supplementary Figure S7), MCP-2 is the only cytokine that appears to be regulated by H4K12ac since inhibition of H4K12ac is resulting in a reduction of MCP-2 production induced by EtOH as shown in Fig. 5 panel b. In Supplementary Figure S7, panel a, a graphical representation of optical density values show treatment with 0.2% EtOH significantly upregulates ICAM-1 (4.71 ± 0.42, p = 0.0007) and IL-10 (5.008 ± 0.89, p = 0.027). While inhibition of H4K12ac by NU9056 (50 nM) significantly upregulates ICAM-1 (7.37 ± 1.16, p = 0.002), IL-6 (4.55 ± 1.21, p = 0.015), IL-10 (4.13 ± 1.03, p = 0.046), IL-15 (4.33 ± 1.84, p = 0.062), IL-16 (2.62 ± 1.02, p = 0.059) and IP-10 (4.15 ± 0.69, p = 0.002) when compared to untreated control. In addition, when the cells were treated with a combination of 0.2% EtOH and NU9056, there was a significant upregulation of ICAM-1 (10.43 ± 2.42, p = 0.011), IL-10 (4.35 ± 0.55, p = 0.023), IL-15 (6.68 ± 1.01, p = 0.0009), IL-16 (1.94 ± 0.35, p = 0.0004) and IP-10 (3.95 ± 0.35, p = 0.0002) when compared to untreated control. Statistical analysis between MDDCs co-treated with 0.2% EtOH + NU9056 versus MDDCs treated with only 0.2% EtOH to reveal a significant upregulation of IL-15 (p = 0.036), RANTES (p = 0.014), TGFβ−1 (p = 0.0008) and TNFα (p = 0.028) shown in Fig. 5 panel a and significant downregulation of MCP-2 (p = 0.016) shown in Fig. 5 panel b. Representative blots for each treatment are included in Supplementary Figure S7, panel b. To further validate the modulation of MCP-2 by H4K12ac, MCP-2 levels were measured by ELISA (Fig. 5 panel c) in cell culture supernatants. The combination of 0.2% EtOH and NU9056 50 nM (321.64 pg/mL ± 13.50, p = 0.03) significantly reduced MCP-2 levels compared to 0.2% EtOH treatment alone (401.75 pg/mL ± 28.22). MCP-2 expression was measured by single cell imaging flow cytometry (Fig. 5 panels d–g) in MDDCs. 0.2% EtOH was able to significantly upregulate percentage of cells expressing MCP-2 (85.8% ± 5.2, p = 0.002) compared to untreated control MDDCs (52.3% ± 2.3). Combination treatment of 0.2% EtOH + NU9056 50 nM also significantly reduced the percentage of cells expressing MCP-2 (29.6% ± 1.18, p = 0.0001) compared to 0.2% EtOH-treated MDDCs. EtOH-treated MDDCs showed significantly higher MFI of MCP-2 (4260 ± 603, p = 0.04) compared to untreated MDDCs (2510 ± 29) while MDDCs treated with 0.2% EtOH + NU9056 50 nM showed significantly lower MFI (1459 ± 297, p = 0.01) compared to 0.2% EtOH-treated cells. Panel f shows an overlay histogram of MCP-2 for isotype (yellow), control (red), 0.2% EtOH (blue), NU9056 50 nM (pink) and 0.2% EtOH + NU9056 50 nM (green). Panel g shows representative single cell images for each treatment. *in silico* analysis using GeneMANIA or Gene Multiple Association Network Integration Algorithm^27^ was carried out to elucidate interactions between KAT5, gene for TIP60 histone acetyl transferase, and NFKB1, which is a common transcription factor associated with alcohol effects^28^ and CCL8 (MCP-2 gene). Results from the *in silico* analysis (Fig. 6 panel a) showed several genes that are associated to KAT5 through physical interaction, gene interaction, or other pathways. KAT5 associates with NFKB1 through physical interactions while CCL8 is co-expressed with NFKBIZ, NFKBIA, RELB and MAP3K8, which are genes that interact directly with NFKB1.

## Discussion

Post-translational modifications of histones are dynamic and are responsible for two broad functions, modifying chromatin state and modulating DNA functions in terms of DNA replication, gene transcription and translation^29^. The goal of this study was to implement two novel techniques: MALDI-FT-ICR MS and single cell imaging flow cytometry as screening tools to delineate histones H3 and H4 post translational modifications in human dendritic cells (DCs) under chronic alcohol stress with an aim of identifying epigenetic markers or epi-drug targets for alcohol use disorders.

Our studies quantifying H3 and H4 (Fig. 1) in untreated and EtOH-treated MDDCs revealed, for the first time, an evident increase in H3 and H4 quantity after treatment with EtOH. Although in the current study nucleosome density was not measured, the detected increase in H3 and H4 may not be a direct correlation towards increase in nucleosome density and this effect may be induced by cell stress due to chronic EtOH treatment as previously shown during extra-nuclear detection of histones and nucleosomes in activated human lymphoblasts as an early event in apoptosis^22^. Recently, Celona *et al*., have demonstrated that histone content is reduced and cells with reduced histones have a more accessible chromatin and increased transcription^30^. Other group has suggested that histone content increases in differentiating embryonic stem cells and that the difference in histone content is an additional hallmark of pluripotency^23,31^. In our study, we observed an increased in histone content which may correlate with the MDDC differentiation stage and maturation effects induced by chronic exposure to EtOH.

Besides an increase in H3 and H4 quantity, our findings also reveal histone H3 and H4 post translational modifications in MDDCs under chronic EtOH treatment. For instance, treatment with 0.2% EtOH for 5 days caused a decrease in H3K4me1, H3K4me3, H3K9me3, H3K36me3 and H3K18ac. In general, H3K9 methylations are mostly associated with gene repression while H3K4 methylations have been mostly associated with gene activation^32,33^. Mono-methylation at H3K27, H3K9, H4K20, H3K79, and H2BK5 are usually related to gene activation; however, tri-methylations of H3K27, H3K9, and H3K79 are related to gene repression^34,35^. Tri-methylation at H3K36 is again associated with active gene transcription^36^ and acetylation at H3K18 is increased in transcriptional start sites and enhancers of active mammalian genes^37,38^. Therefore, based on current literature recently reviewed^9^, our study suggests a state of both activation and repression of gene transcription based on the H3 modifications due to chronic alcohol treatment in MDDCs. Previous reports of chronic alcohol exposure and H3 modification in liver nuclear extracts from rats chronically fed with alcohol showed an increased acetylation at H3K9 and H3K27^39^. Chronic intermittent alcohol (CIE) exposure also showed an increase in histone acetylation at H3K9 while a decrease in H3K9 methylation during CIE removal in mouse primary cortical neurons^40^. Consistent with previous literature, our study also shows a decrease in H3K9 tri-methylation after chronic treatment of MDDCs with 0.2% EtOH. Overall, there was a general decrease in methylation at different H3 lysine residues, which may be due to the chronic EtOH effects on these immune cells.

MDDCs chronically treated with 0.2% EtOH expressed lower levels of H4K5ac, H4K8ac, H4k20m2, H4K20m3, H4R3m2 and H4ser1P with significant upregulation of H4K12ac. According to previous research, acetylation at H4K5, H4K8 and H4K12 are elevated in promoters of transcriptionally active genes, whereas H4K20me3 is associated with repressed genes^38^. While H4R3m2 has previously been shown to be a repressive mark^41^. In our study, altered H4 modifications due to chronic alcohol treatment also show a combination of gene activation and repression in MDDCs. Consistent with our findings, previous literature also suggests an increase in H4K12ac in the context of alcohol and oxidative stress in adolescent rats due to intermittent alcohol administration for two weeks^42^. In another report, a significant increase in H4K12ac was seen in monocytes from transgenic Alzheimer’s rats which also resulted in the increase of inflammatory cytokines like MIP-2 and TNFα^43^. Additionally, H4K12 and H3K18 acetylations have been indicated as prognostic markers for pancreatic cancer showing a high correlation score with tumor stage^44^. Extrapolating from previous reports of H4K12ac as prognostic marker for Alzheimer’s^44^ and pancreatic cancer^44^, and from our current findings demonstrating an increase in H4K12ac after chronic alcohol exposure, it can be concluded that upregulated H4K12ac could serve as an important epigenetic marker during chronic alcohol abuse.

Recent progress in epigenetic histone modification analysis support the use of mass spectrometry for clinical applications^45,46^. Therefore, using MALDI-FT-ICR-MS we were also able to confirm the ELISA, single cell imaging flow cytometry, and western blotting results showing EtOHinduced an increase in H4K12ac in human MDDCs. It is relevant to point out that although all the techniques used demonstrated a significant increase in H4K12ac after alcohol treatment, there was variability in the increased amount. For instance, ELISA results showed a four-fold increase in H4K12ac (Fig. 1 panel c) and the validation methods using immunoblotting (Fig. 2), MALDI-FT-ICR-MS (Fig. 3), and single cell imaging flow cytometry (Fig. 4) were consistent in demonstrating an increase in H4K12ac; although with a less profound effect. Single cell imaging flow cytometry data demonstrated the ability of alcohol to significantly increase the percentage of H4K12ac positive cells (Fig. 4 panel c); however, the MFI of intracellular H4K12ac expression per cell (Fig. 4 panel d) was not significantly increased. This could be due to the variability of the amount of acetylation per cell since not all histones are acetylated at the same rate and global levels of histone acetylation can be modulated due to different variables including pH^47^; however, the reason why cells regulate histone acetylation levels after EtOH exposure and the mechanisms of action still remain to be elucidated.

Furthermore, this study highlights the use of two novel approaches, MALDI-FT-ICR MS and single cell imaging flow cytometry as screening tools to detect PTMs in human immune cells. Additionally, this study reports, for the first time, the ability of NU9056, a recently identified Tip60/HAT specific inhibitor^19^, to block the alcohol-induced effect on H4K12 acetylation in primary culture of human MDDCs. Results from H4K12ac quantification and inflammatory cytokine arrays from supernatants of cells under treatment with 0.2% EtOH, or NU9056, or both, show NU9056 reduces H4K12 acetylation compared to control (Fig. 4) and when H4K12ac is reduced there is an evident upregulation of cytokines like ICAM-1, IL-6, IL-10, IL-15, Il-16 and IP-10 when compared to control (Supplementary Figure S7). Surprisingly, NU9056 in combination with 0.2% EtOH treatment enhanced the effects of EtOH on cytokine production by upregulating the secretion of IL-15, RANTES, TGFβ−1 and TNFα compared to EtOH 0.2% while blocking the secretion of MCP-2 compared to 0.2% EtOH treatment, suggesting a partial inflammatory role of H4K12ac.

Even though TIP 60 has been reported to be involved in acetylation of H4K12^17,18^, it is also an acetyl transferase for other acetylation sites on H4^16^. Therefore, NU9056 being a TIP60 inhibitor, may be modulating other acetylation sites which affect cytokine expression and result in other functional effects on MDDCs. Overall, the functional effects of NU9056 and/or EtOH resulting in increased inflammation may not solely be mediated through H4K12ac, since modulation in other acetylation sites may also be involved. HAT inhibitors like anacardic acid have been reported to be protective in function against alcohol-induced H3K9 hyper-acetylation, Gata4, α-MHC, cTnT over-expression by inhibiting the binding of certain HATs to the promoter region of these genes^48^. NU9056 has recently been shown to have a protective role in mouse neurons by reducing increased cyclic phospholipase mRNA expression due to oxygen-glucose deprivation injury^49^. In regards to inflammation, epigenetic regulation of pro-inflammatory cytokines in PBMCs from post-traumatic stress disorder (PTSD) patients show evidence of the role of epigenetic regulation during inflammation^50^. In the current study, although NU9056 significantly lowers MCP-2 secretion induced by EtOH, surprisingly the effect of EtOH on inflammatory cytokines (IL-15, TGFβ-1 and TNFα)^51^ and pro-inflammatory chemokine (RANTES)^52^ is enhanced by NU9056.

A possible explanation for the modulation of inflammation induced by NU9056 and/or EtOH treatments is toxicity as a result of chronic exposure; therefore, to rule out cellular toxicity as an aberrant side effect cell viability (Supplementary Figure S5) and apoptosis/ necrosis assays (Supplementary Figure S6 panels a–c) were performed in MDDCs untreated and chronically treated with NU9056 and/or EtOH. Although the results demonstrate there is about 20% apoptosis among untreated and treated cells as detected by 7-AAD and annexin V staining (Supplementary Figure S6 panels c), there are no significant differences in apoptosis or necrosis between the treatments. Furthermore, LDH release in cell culture media was also measured and confirm similar levels among untreated and treated cells (Supplementary Figure S6 panel d). Therefore, it is unlikely that the differential modulation in cytokine release observed is solely due to cellular toxicity caused by NU9056 and/or EtOH.

Another possible explanation for the enhancement of inflammatory cytokines is interactions between EtOH and NU9056; as we have previously reported in the case of co-treatment of MDDCs with mocetinostat and EtOH, which resulted in the exacerbation of production of oxidative stress related genes^8^. Histone modifications and inflammatory cytokine regulation have been correlated with oxidative and cellular stress as previously reported^53–55^. Therefore, cellular stress could be the cause of altered histone modifications which may be helping the cells to maintain homeostasis by altering transcription of inflammatory genes. Although we did not measure reactive oxygen species (ROS), ROS signaling is crucial during innate and adaptive immunity and the effect of post-translational modifications on proteins related to ROS signaling may be playing a role on the immunomodulation observed after chronic alcohol exposure of MDDCs such as in the case of hypoxia inducible factor (HIF-1α)^56^ or elevated mitochondrial superoxide (O_2_^−^) levels, which has been shown to disrupt normal T-cell development impairing adaptive immune function under other stress conditions such as influenza challenge^57^. Furthermore, chronic ethanol exposure may be modulating the ability of DCs to activate co-stimulatory molecules and induce cytokine response as in the case of chronic ethanol feeding in mice, which limits the ability of DCs to stimulate T cell proliferation^58,59^.

In summary, H4K12ac negatively regulates IL-15, RANTES, TGF-β1 and TNF-α while positively regulating MCP-2 (Fig. 6 panel b). Even though typically histone acetylation is related to increased gene transcription and positive regulation, literature also suggests, the process may be more complicated involving transcription factors, other histone modifications and chaperones and can also cause negative regulation or reduction in gene transcription^60^. The *in silico* analysis further throws light on the gene interaction pathways between MCP-2 (CCL8), KAT5 (TIP60 histone acetyl transferase) through NFκB, which is a common transcription factor associated with alcohol effects^61^. KAT5 directly interacts with NFKB1 while CCL8 is co-expressed with NFKBIZ, NFKBIA, RELB and MAP3K8, demonstrating that the alcohol effect on H4K12ac may be mediated through NKκB. In addition, NFKBIA is an NFκB inhibitor which inhibits the activity of dimeric NF-kappa-B/REL complexes^62^, suggesting a possible mechanism by which H4K12ac modulates MCP2/CCL8 expression. To further analyzed the signaling mechanisms depicted in the *in silico* analysis, it may be worth directly targeting H4K12ac with siRNA or CRISPR Cas9 techniques to block TIP60 histone acetyl transferase as previously described^63,64^.

In conclusion, this is the first study to provide evidence of H3 and H4 modification landscape due to chronic alcohol exposure in human MDDCs. It also brings into light H4K12ac as an epigenetic marker for MDDCs under chronic alcohol stress. We also show TIP60/HAT inhibitor, NU9056, was able to block the effects of chronic EtOH on H4K12ac; however, this inhibition of H4K12ac resulted in an increase of cytokines with a significant downregulation of MCP-2. Similar to our findings, Plagg and colleagues have reported an increased in TNFα along with other cytokines to be correlated with an increased in H4K12ac in monocytes from transgenic Alzheimer’s rats^43^. The ability of NU9056 to inhibit MCP-2 upregulation by EtOH may serve as a protective mechanism against inflammation due to chronic EtOH exposure further confirming the functional role of H4K12ac as an inflammation regulator since MCP-2 is functionally important as a chemokine that attracts eosinophils, basophils, monocytes and T cells^65^.

Besides the implementation of novel screening tools, this study is of significance as it elucidates the mechanisms behind alcohol effects in the immune system, and it contributes to the current research priorities of the National Institute on Alcohol Abuse and Alcoholism (NIAAA), where epigenetics and precision medicine have become an important factor to be considered when studying the molecular mechanisms behind alcohol effects.

## Materials and Methods

### MDDCs Isolation

Human buffy coats from healthy anonymous blood donors were purchased from the community blood bank (One Blood, Miami, FL, USA). Human blood studies in Dr. Agudelo’s lab have been reviewed and approved by the Institutional Review Board of FIU, IRB protocol approval # IRB-13-0440. All methods were performed according to institutional guidelines. Buffy Coats are commercially available and each buffy coat represents one N in our experiments. Monocytes were isolated from buffy coats and MDDCs were differentiated as previously described by us^8^.

### Treatments

MDDCs were treated with NU9056 (#4903, Tocris, USA.) 50 nM and/or 0.1% (~20 mM) alcohol (Ethanol or EtOH) (catalog #E7023, Sigma–Aldrich, St. Louis, MO, USA) or 0.2% EtOH (~40 mM) for 5 days, which are equivalent to the physiological blood alcohol concentrations (BAC) of 100 mg/dL and 200 mg/dL respectively, and are close to the legal limit for driving under intoxication of 0.08% (80 mg/dL) and corresponds to BAC levels found in chronic alcohol users (200 mg/dL)^66^. Our usage of 0.2% alcohol is also supported by literature^67^. Alcohol treatments were replenished in full every 24 hours and media changed every 48 hours. Alcohol treated cells were kept in a separate incubator (New Brunswick™ Galaxy® 48 R) humidified with alcohol matching the concentration in culture conditions, whereas control cells were kept in a separate incubator (VWR® symphony™ Air-Jacketed CO2 Incubator).

### Viability and Toxicity assays

Trypan blue assay was carried out as previously described by us^8^. XTT assay was carried out using XTT cell proliferation assay kit (#35-1011 K, ATCC), apoptosis assays were carried out using PE Annexin V apoptosis detection kit (#559763, BD Biosciences) and LDH activity measurement in cell culture medium of cells was carried out using LDH assay kit (#AB102526, ABCAM) following manufacturers protocol.

### Histone extraction and total H3, H4 and H4K12ac quantification

Total histones were extracted from MDDCs using histone extraction kit (EpiQuik Total Histone Extraction Kit, #OP-0006, Epigentek) and were quantified with Bradford reagent (Bio-Rad Protein Assay Dye Reagent Concentrate, #5000006) using spectrophotometer (VWR® Spectrophotometers, UV-Vis Scanning UV-3100PC). ELISA-based EpiQuik quantification kits were used to measured total H3 (EpiQuik Total Histone H3 Quantification Kit, #P-3062-96, Epigentek), total H4 (EpiQuik Total Histone H4 Quantification Kit, #P-3072-96, Epigentek) and total H4K12ac (EpiQuik Global H4K12ac Quantification Kit, #P-4028-96, Epigentek). Quantity values were calculated using manufacturer’s instruction which accounts for protein amount used and based on a standard curve generated for each assay using known amounts of H3, H4 or H4K12ac protein respectively. 500 ng of histone extracts were assayed for each sample. To standardize these data, raw quantification values were then converted into percent of control for each experiment and data for all independent experiments were combined. The formula [Amount (ng/mg protein) = {OD (sample − blank) × 1000}/(Protein (µg)* × slope)] was used for H3, H4 and H4K12ac quantification. Slope is derived from standard curves as shown in Supplementary Figure S1. As they are human samples, to account for variability, amount of H3 and H4 obtained was converted to % of control for each experiment then H3 and H4 was represented in one graph.

### H3 and H4 modification detection and analysis by ELISA

These modifications have been selected in our study based on previous findings^8,68–70^. Histone modification studies were carried out using H3 modification multiplex array (EpiQuik Histone H3 Modification Multiplex Assay Kit, #P-3100-96, Epigentek) and H4 modification multiplex array (EpiQuik Histone H4 Modification Multiplex Assay Kit, #P-3102-96, Epigentek). Equal amounts of protein were added among samples in each independent experiment, however, overall protein concentration range from 100 to 200 ng. H3 modifications were calculated according to manufacturer’s instructions which also accounts for protein amounts and the final values for each modification were presented as percentage over untreated control.

### H4K12ac detection by immunoblotting

2 µg of total histone extracts were electrophoresed on a 4–20% polyacrylamide gel (catalog# 456-8094, BIORAD). Western blot was performed as previously described by us^7^. Antibodies used were rabbit anti-human-H4K12ac antibody (catalog# 19-595 EMD Millipore) (1:2000) and secondary antibody, anti-rabbit (1:2000) (catalog# 2-348 EMD MILLIPORE), anti-human H4 (1:10,000) (#MA5-14816, Thermo Scientific™) and secondary anti-mouse (1:10,000). Films were analyzed using Image J software.

### H4 peptide mapping of H4 by mass spectrometry: Gel Preparation

36 µg of total histone extracts pooled from untreated and 0.2% EtOH treated MDDCs were run on 4–20% SDS-PAGE. Histone H4 bands observed at around 14-kDa were excised and placed in clean, plastic vials prior to in-gel digestion with trypsin. **In-gel tryptic digestion** was conducted utilizing the Thermo Scientific digestion kit (#89871) following manufacturers protocol. Extraction of peptides was performed by adding 10 μL of 1% Formic Acid (FA) to the gel band. Thereafter, the aliquot was combined with the gel peptide extract, and used for Matrix Assisted Laser Desorption Ionization-Fourier Transform Ion Cyclotron Resonance Mass Spectrometry (MALDI-FT-ICR MS) analysis. MALDI mass spectrometry analysis was conducted on a 7 T Bruker Solarix FT-ICR MS instrument (Bruker Daltonics Inc., Billerica). The instrument and sample spotting technique are similar to previously described^71^. The mass spectra were internally calibrated with a 0.01 ng/mL solution of Sodium Trifluoroacetate (NaTFA) utilizing 10 calibration points enclosing the mass range between 300 to 1600. Mass spectra were acquired with 50 co-added scans, with 200 laser shots at 500 Hz and at 4 MW. Considering that MALDI matrix peaks are quite abundant below 700 Da, the database search excluded known CHCA matrix peaks between 300 and 700 Da from the peak list submitted for peptide mass fingerprinting. Data was processed using the Biotools Protein Viewer and Sequence Editor software (Bruker Daltonics Inc., Billerica). Spectra were normalize to commonly observed non-acetylated (e.g., 1180.6216).

### H4K12ac and MCP-2 immunostaining and analysis by single cell imaging flow cytometry

1 × 10^6^ cells were stained with primary anti-human H4K12ac-polyclonal (# 07-595 EMD Millipore) and secondary anti-rabbit Fluorescein isothiocyanate (FITC)-labelled antibody (catalog #AP187F, Millipore). Rabbit isotype control antibody (catalog #02-6102, Thermo Fisher) was also used. Intra-nuclear staining for H4K12ac was carried out using transcription factor buffer kit (catalog #562574, BD Biosciences) following manufacturer’s protocol. For MCP2 staining, MCP2-FITC labeled antibody (#NB120-10391F, Novus) was used following manufacturers protocol for the fixation/permeabilization kit (#55028, BD Biosciences). Cells were first stained with fixable viability dye eFLUOR®450 (Cat# 18-0863, eBioscience) to gate on live cell population during image acquisition. DAPI (4′,6-Diamidino-2-Phenylindole, Dihydrochloride) was used in co-localization studies. Image acquisition by Amnis® FlowSight® Imaging Flow Cytometer and analysis by IDEAS® image software was carried out as previously described by us^72^. For each experiment, from all events collected, FITC positive cells were gated from single cells.

### Cytokine array and MCP-2 ELISA

The expression of 48 inflammatory cytokines was analyzed according to manufacturer instructions with the RayBiotech inflammation arrays (#AAH-INF-3-8, RayBiotech, Norcross, GA, USA) as previously described by us^25,26^. Briefly, supernatants from cells after 5 day post-treatment with NU9056, 0.2% EtOH and 0.2% EtOH + NU9056 and untreated control were assayed for the experiment. MCP-2 ELISA was carried out using MCP-2 ELISA kit (#ELH-MCP2-1, RayBiotech) as per manufacturer’s instructions.

### *In silico* Analysis

Target cytokines identified with the cytokine array and ELISA were further analyzed *in silico* using the GeneMania prediction server (University of Toronto) for collating gene and pathway interactions.

### Statistics

All graphical and statistical analysis were carried out either in Microsoft excel or Graph Pad Prism software (GraphPad Prism software, La Jolla, CA). IDEAS® image analysis software was used for single cell imaging studies. Comparisons between groups were performed using t-test or two-way ANOVA and Dunnett’s Multiple Comparison post-test. Differences were considered significant at p ≤ 0.05. Data are expressed as mean ± SEM.

## Electronic supplementary material

Supplementary figures
