# Publisher Correction: Novel detection of post-translational modifications in human monocyte-derived dendritic cells after chronic alcohol exposure: Role of inflammation regulator H4K12ac

**DOI:** 10.1038/s41598-017-16204-9

**Published:** 2017-11-21

**Authors:** Tiyash Parira, Gloria Figueroa, Alejandra Laverde, Gianna Casteleiro, Mario E. Gomez Hernandez, Francisco Fernandez-Lima, Marisela Agudelo

**Affiliations:** 1 Department of Immunology, Herbert Wertheim College of Medicine, Florida International University, Miami FL, 33199 United States; 2 Advanced Mass Spectrometry Facility, Department of Chemistry and Biochemistry, Florida International University, Miami FL, 33199 United States

*Scientific Reports* **7**:11236; doi:10.1038/s41598-017-11172-6; Article published online 11 September 2017

In the original version of this Article, Tiyash Parira and Alejandra Laverde were incorrectly affiliated with ‘Advanced Mass Spectrometry Facility, Department of Chemistry and Biochemistry, Florida International University, Miami, FL, 33199, United States’. The correct affiliation is listed below:

Department of Immunology, Herbert Wertheim College of Medicine, Florida International University, Miami, FL, 33199, United States.

This error has now been corrected in the PDF and HTML versions of the Article.

