# Supplementary material for: Novel detection of post-translational modifications in human monocyte-derived dendritic cells after chronic alcohol exposure: Role of inflammation regulator H4K12ac
Source: Sci Rep. 2017 Sep 11;7:11236. doi: 10.1038/s41598-017-11172-6 (PMC5593989; doi:10.1038/s41598-017-11172-6)
Supplement: Supplementary file 1 — Supplementary figures [file 41598_2017_11172_MOESM1_ESM.pdf]

Supplementary DATA

Novel detection of post-translational modifications in human monocyte-derived dendritic cells after chronic alcohol exposure: Role of inflammation regulator H4K12ac.

Tiyash Parira, M.S. [1], Gloria Figueroa, B.S. [1], Alejandra Laverde[1], Gianna Casteleiro[2], Mario E. Gomez Hernandez, Ph.D. [3], Francisco Fernandez-Lima, Ph.D. [3], Marisela Agudelo, Ph.D. [1]*

***Corresponding Author:**

Marisela Agudelo, Ph.D.

Phone: 305-348-6503

Fax: 305-348-1109

magudelo@fiu.edu

Affiliation for all authors:

1. Department of Immunology, Herbert Wertheim College of Medicine, Florida International University, Miami, FL 33199.
2. Department of Human and Molecular Genetics, Herbert Wertheim College of Medicine, Florida International University, Miami, FL 33199.
3. Advanced Mass Spectrometry Facility, Department of Chemistry and Biochemistry, Florida International University, Miami, FL 33199.

H3

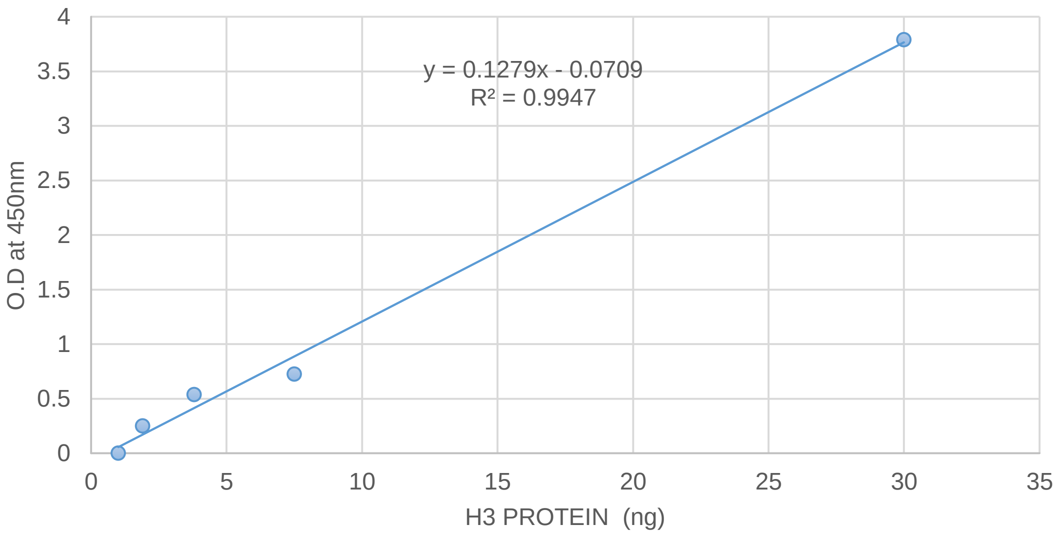

H4

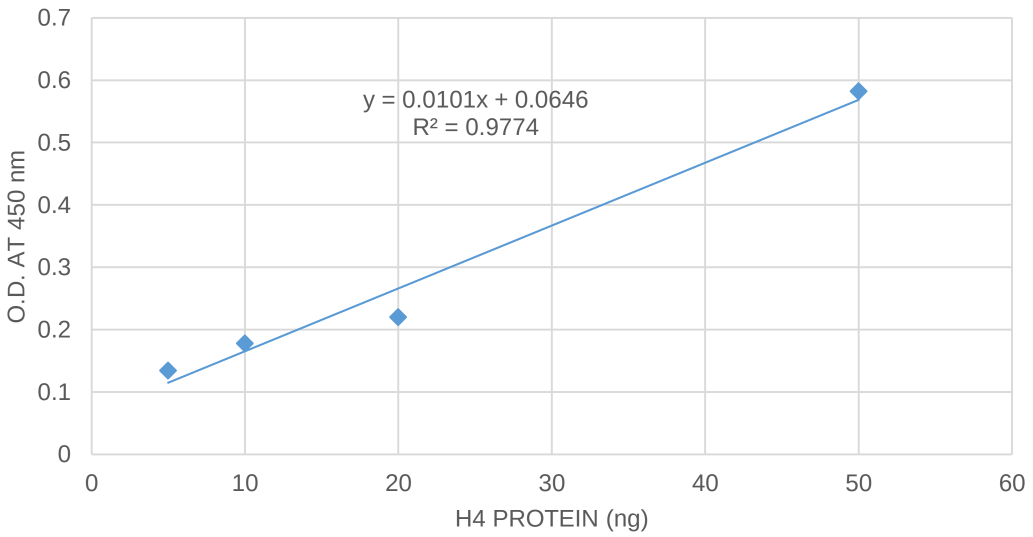

Supplementary Figure S1 : representative standard curves for H3 and H4 quantification experiments. From the respective standard curves, using slope and input histone extract amounts, H3 and H4 amounts were calculated and changed to percent of control based on manufacturer's recommendations. Each experimental plate was carried out with its respective standards.

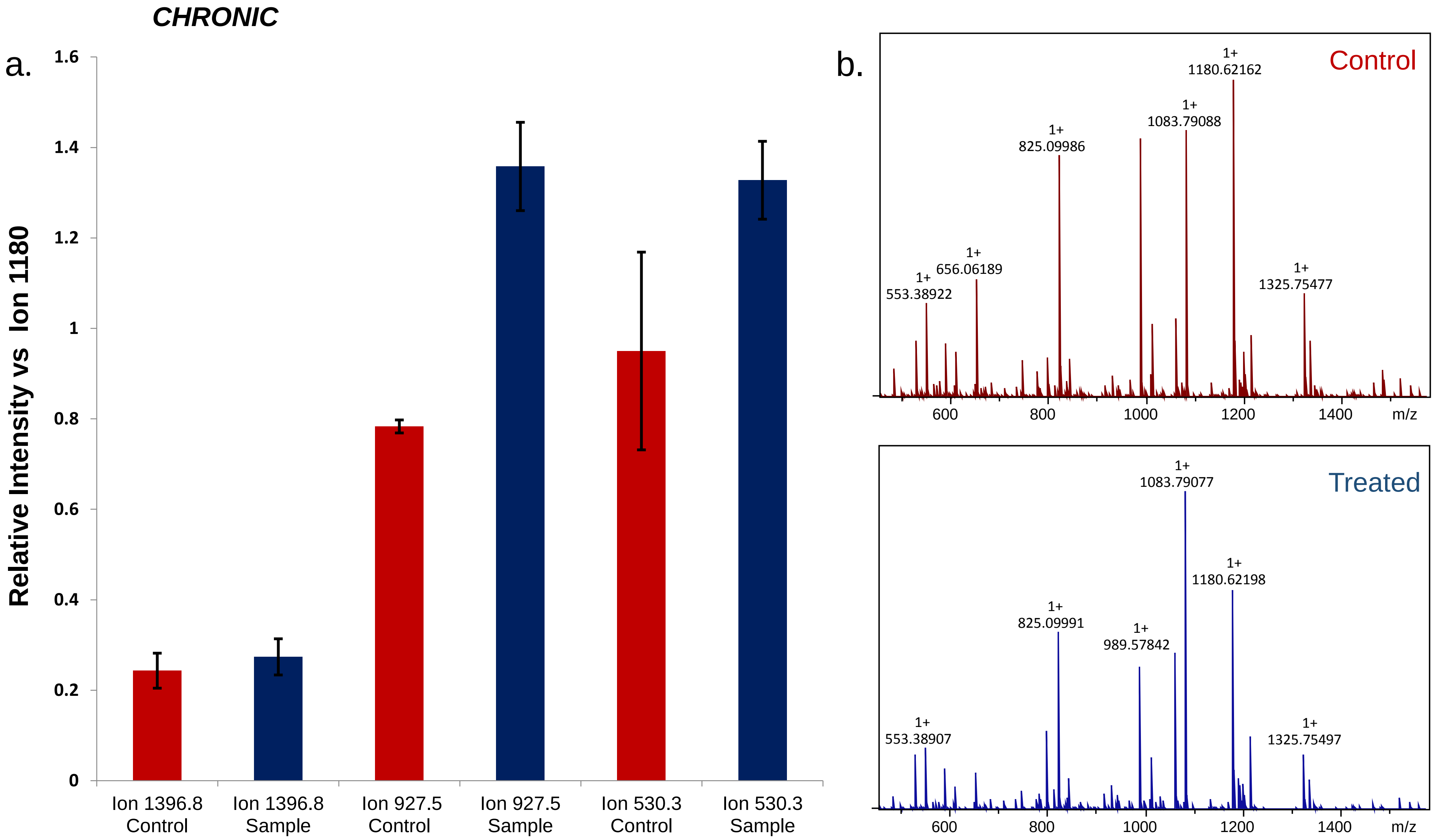

Supplementary Figure S2: Panel a shows relative abundances of 14-18 GGAKR, 10-18 GLGKGGAKR and 5-18 GKGGKGLGKGGAKR peptides in the control and chronically treated samples. Spectra were normalized to the common non-acetylated *m/z* 1180.6216 signal. Error bars obtained from the standard deviation of triplicate sets. Panel b depicts the full mass spectra for the untreated control and MDDCs treated chronically with 0.2% EtOH.

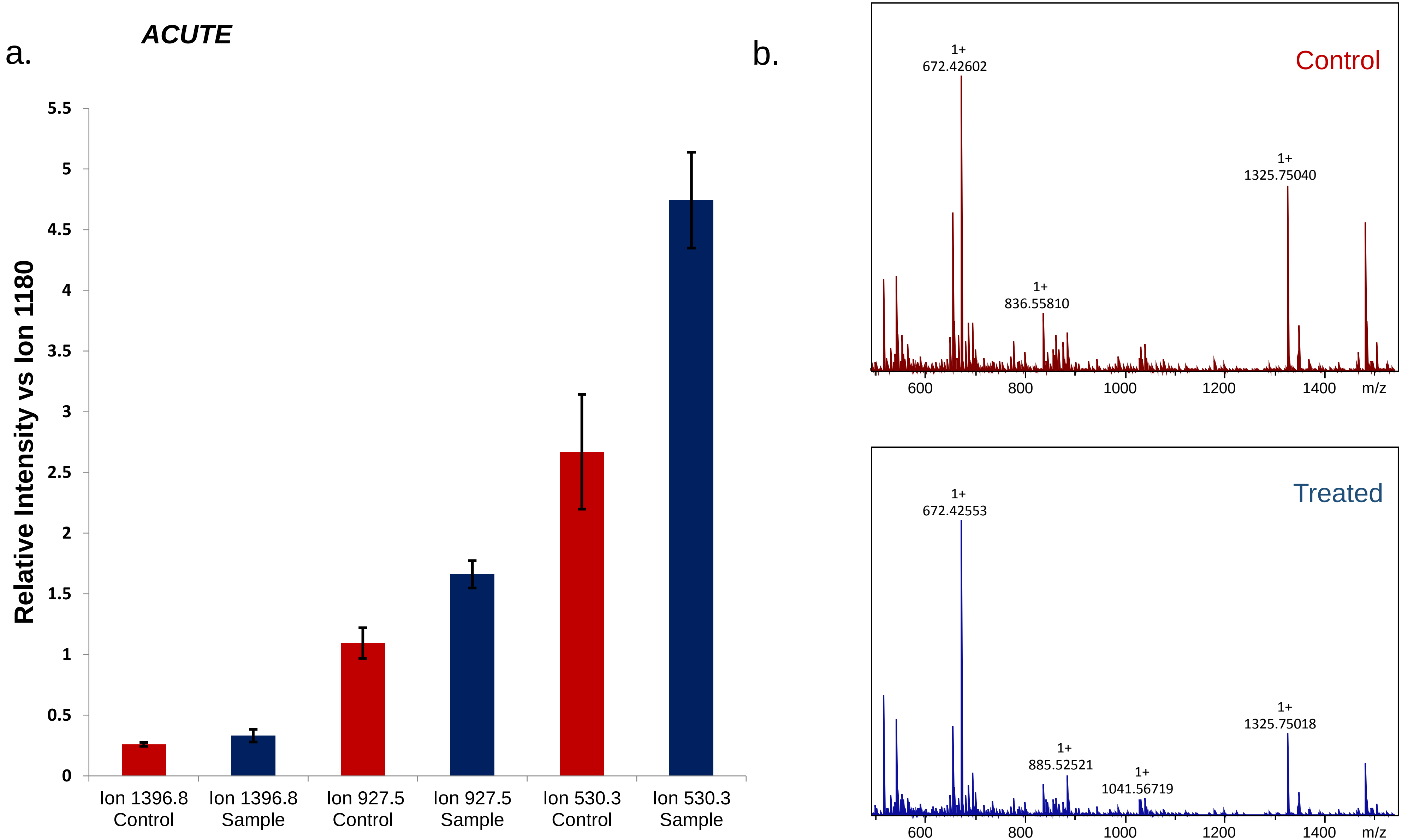

Supplementary Figure S3: Panel a shows relative abundances of 14-18 GGAKR, 10-18 GLGKGGAKR and 5-18 GKGGKGLGKGGAKR peptides in the control and acutely treated samples. Spectra were normalized to the common non-acetylated *m/z* 1180.6216 signal. Error bars obtained from the standard deviation of triplicate sets. Panel b depicts the full mass spectra for the untreated control and MDDCs treated acutely with 0.2% EtOH.

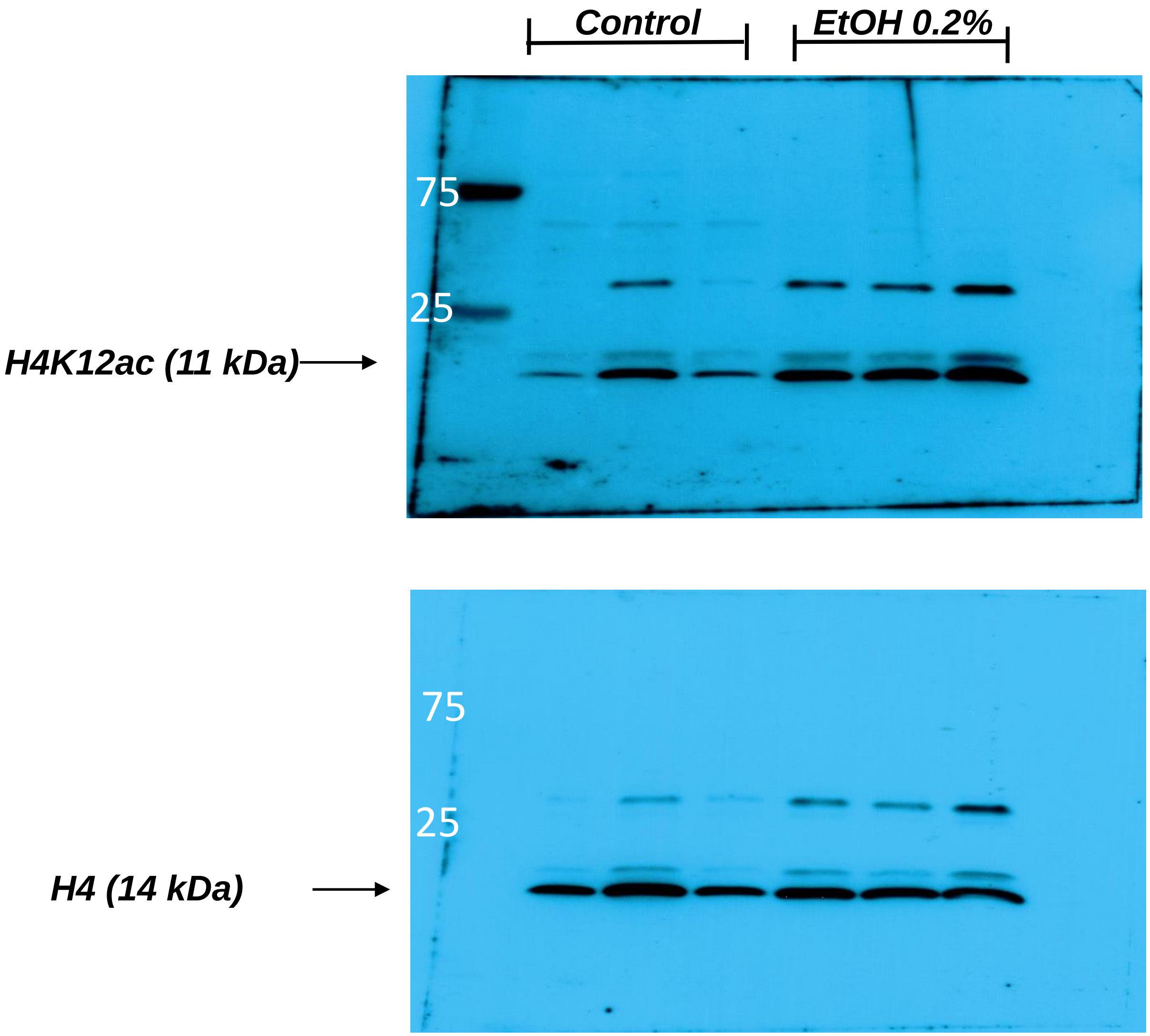

Supplementary Figure S4: Full blots for Figure 2a depicting H4K12ac (11 kDa) and H4 (14 kDa) for untreated control and MDDCs treated chronically with 0.2% EtOH.

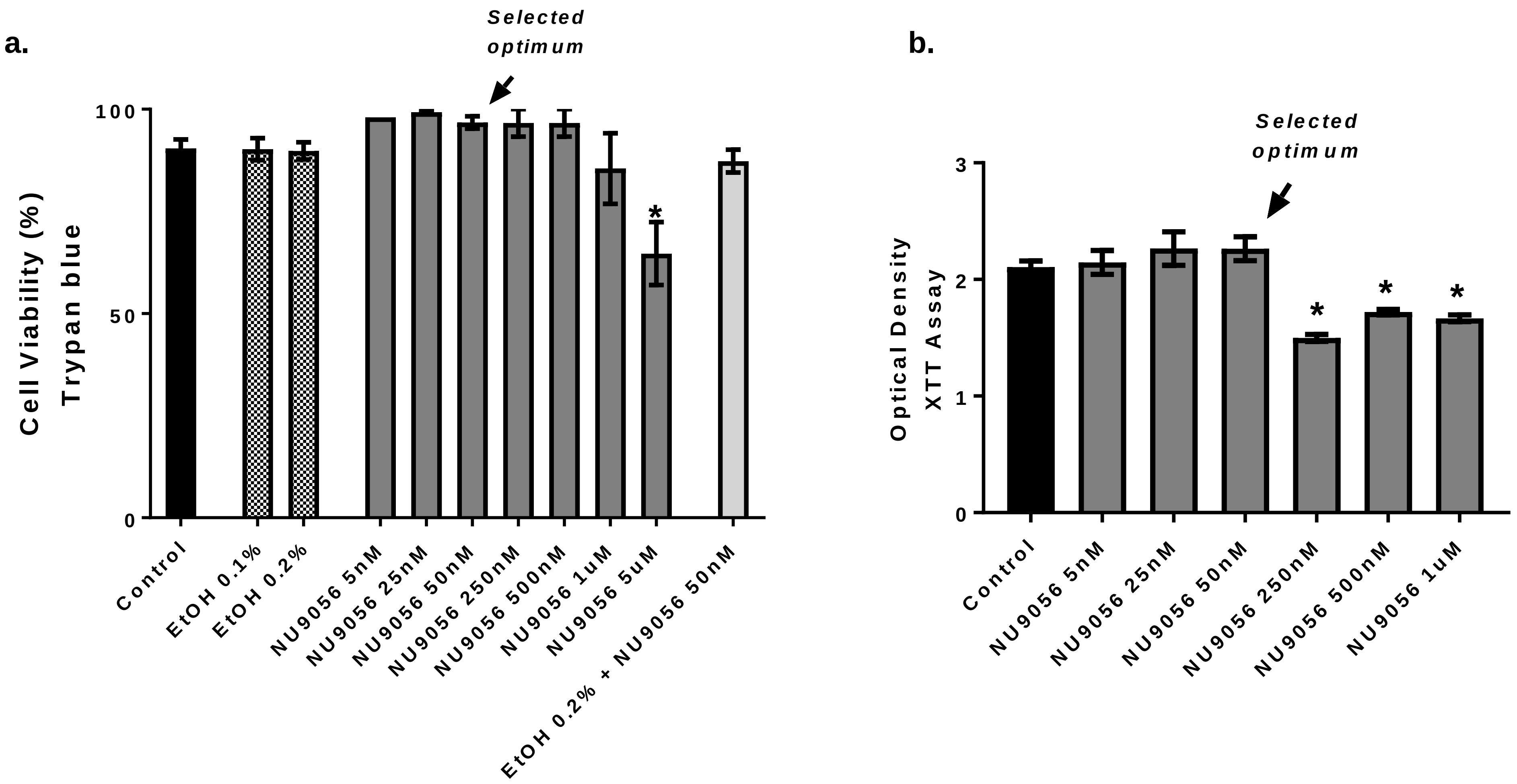

Supplementary Figure S5: Panel a: Viability of cells under all treatments and control were measured using Trypan blue exclusion method where MDDCs were stained with 0.4% trypan blue dye. Live and dead cells were counted using an automated cell counter. Data represented show % viability values. An arrow on NU9056 50nM indicates the chosen concentration for the compound. Significant difference between treatment and control was analyzed by student's t-test. Viability of cells after treatment with 5 µM NU9056 was significantly lower than control ($p=0.001$). There were no significant differences in the other treatments when compared to control. **Panel b:** Based on trypan blue viability results, we selected NU9056 concentrations ranging from 5nM to 1uM and carried out XTT assay following manufacturer's protocol. Results show NU9056 is significantly toxic and altering metabolic activity in MDDCs compared to untreated control MDDCs at concentrations 250 nM, 500nM and 1uM ($p < 0.001$). NU9056 50nM is the optimum non-toxic concentration that can be used in MDDCs under chronic treatment.

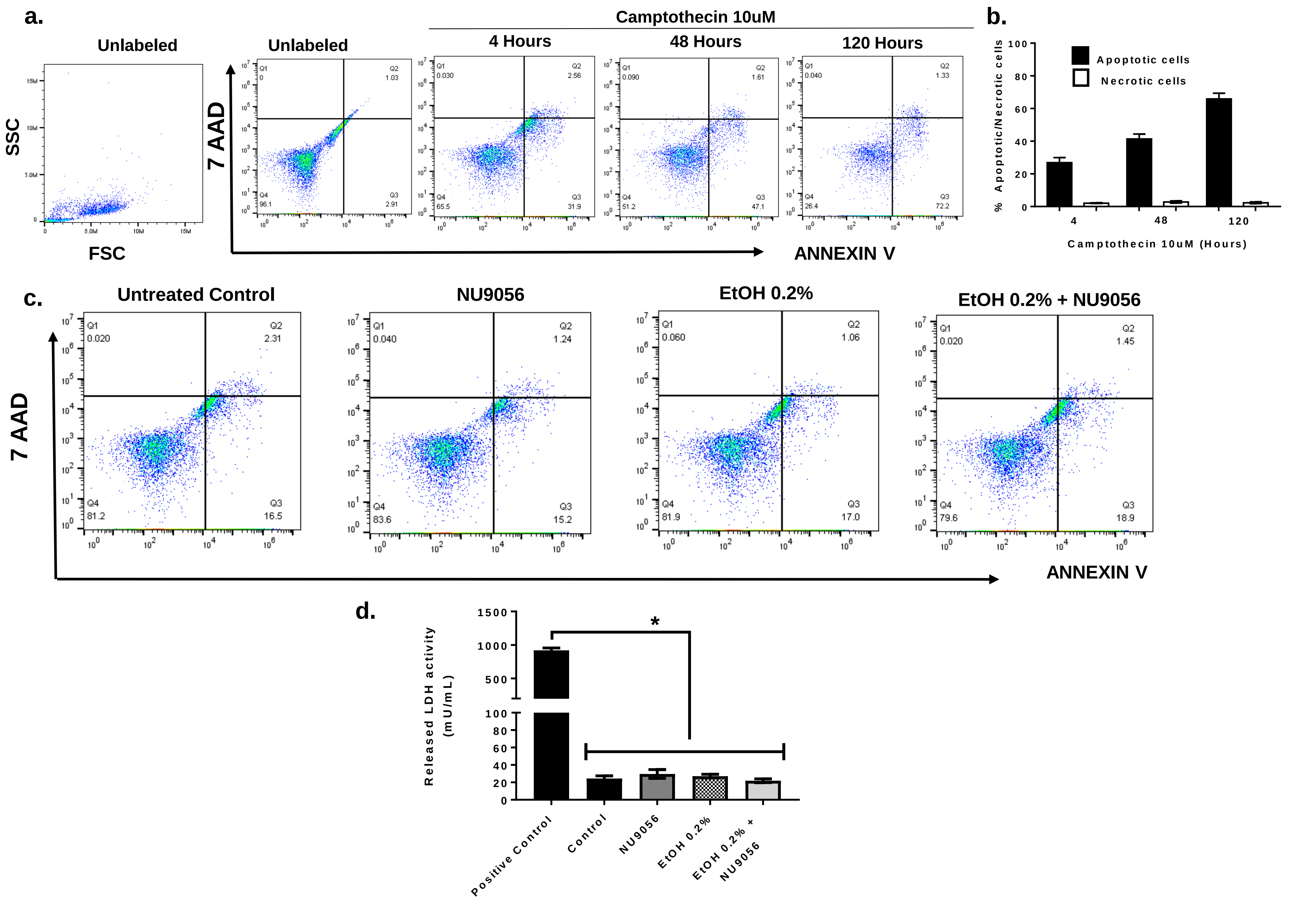

a.
Camptothecin 10uM
Unlabeled
SSC
FSC
Unlabeled
4 Hours
48 Hours
120 Hours
7 AAD
ANNEXIN V
Q1 0
Q2 1.03
Q4 96.1
Q3 2.91
Q1 0.030
Q2 2.56
Q4 65.5
Q3 31.9
Q1 0.090
Q2 1.61
Q4 51.2
Q3 47.1
Q1 0.040
Q2 1.33
Q4 26.4
Q3 72.2
b.
Apoptotic cells
Necrotic cells
% Apoptotic/Necrotic cells
Camptothecin 10uM (Hours)
4
48
120
c.
Untreated Control
NU9056
EtOH 0.2%
EtOH 0.2% + NU9056
7 AAD
ANNEXIN V
Q1 0.020
Q2 2.31
Q4 81.2
Q3 16.5
Q1 0.040
Q2 1.24
Q4 83.6
Q3 15.2
Q1 0.060
Q2 1.06
Q4 81.9
Q3 17.0
Q1 0.020
Q2 1.45
Q4 79.6
Q3 18.9
d.
Released LDH activity (mU/mL)
*
Positive Control
Control
NU9056
EtOH 0.2%
EtOH 0.2% + NU9056

Supplementary Figure S6: Apoptosis/necrosis assays **Panel a:** Optimization of positive control (10uM camptothecin) was carried out at least twice and treated for 4, 48 and 120 hours. Representative scatter plots are shown in panel a followed by graphical representation of apoptotic and necrotic cells in Panel b. **Panel b:** At 4 hrs, there is about 26% apoptotic and 2% necrotic cells, at 48 hrs about 42% apoptotic and 2.8% necrotic cells and by 120 hrs there was 63% apoptotic and 3% necrotic cells. **Panel c**: representative scatter plots showing staining with 7-AA (y-axis) and annexing V (x-axis) for untreated control and treated MDDCs. Results show apoptotic cells range around 18 – 20 % among all treatments. Additionally, camptothecin induced significantly higher apoptosis compared to control, NU9056 50nM, 0.2% EtOH and 0.2% EtOH+NU9056 50nM ($P<0.01$). **Panel d:** Lactate dehydrogenase (LDH) activity was also measured in cell culture medium from cells untreated or treated with EtOH 0.2%, NU9056 50nM or both. Panel d shows all treatments show about 21-30 mU/mL LDH activity in cell culture medium, however no significant difference lies between treatments. Manufacturer provided positive control (lyophilized LDH) showed significantly ($P<0.001$) higher LDH activity compared to all treatments.

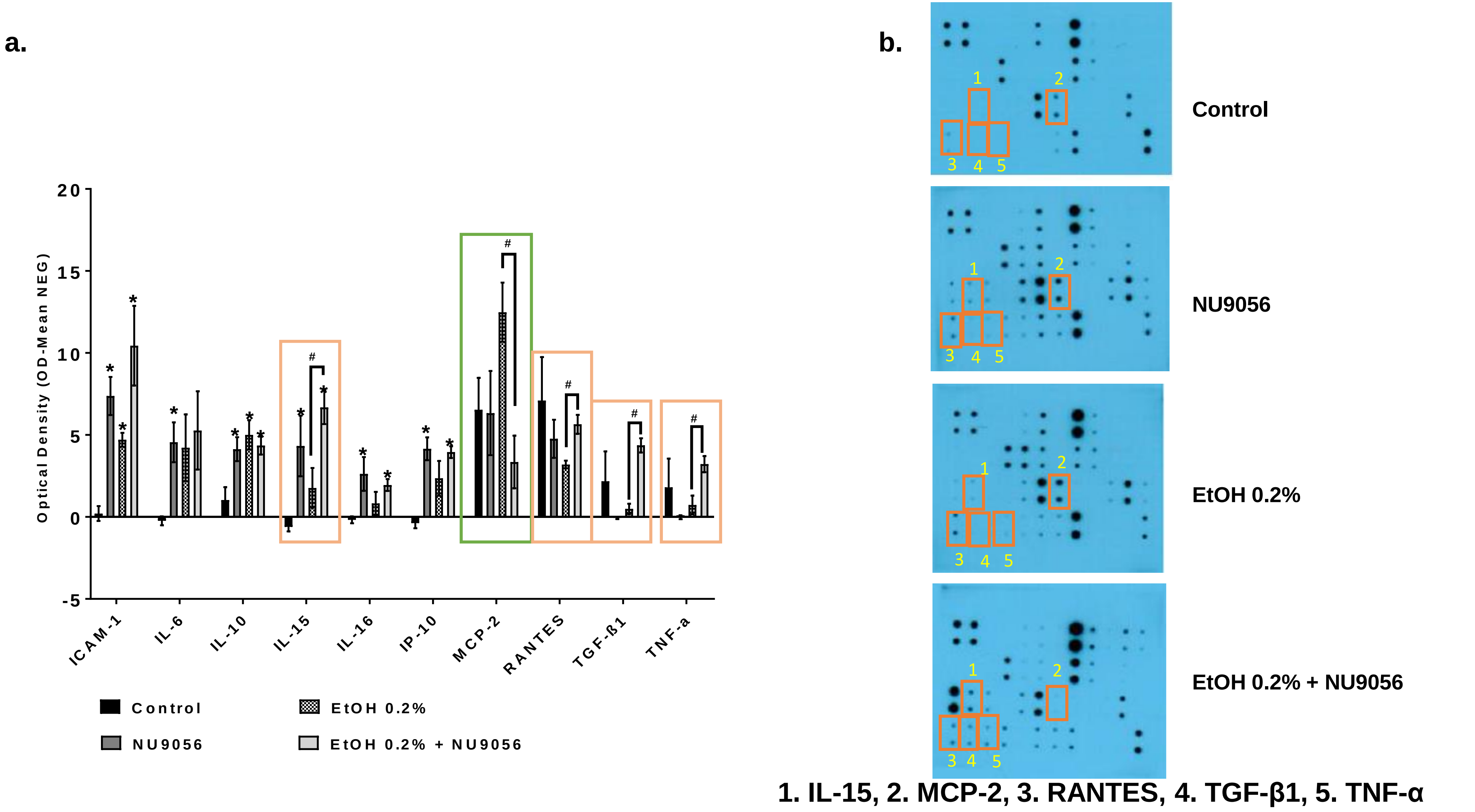

Supplementary Figure S7: After 5-7 days of differentiation, MDDCs were treated with NU9056 50nM, 0.2% EtOH or both for 5 days. Supernatants collected were analyzed for 48 inflammatory cytokines and chemokines. Array blots were analyzed using ImageJ software. **Panel a** shows graphical representation of optical density (OD) values post background correction (subtracting of mean negative from OD) ± SEM. Represented data is from 3 blots for untreated control, 2 blots each for treatment with 0.2% EtOH alone, treatment with NU9056 alone, and treatment with 0.2% EtOH plus NU9056. Cytokines chosen to be presented in the graph were selected on the basis of fold change (2 folds or more) compared to control. Boxes highlight cytokines shown in Figure 5. Each cytokine is detected in duplicates within each blot. Statistical differences were calculated using student's t-test when individually compared to untreated control and indicated with a * (compared to control) and # (between treatments) for significant p value. Two-way ANOVA was carried out to test for interaction ($p = 0.027$). **Panel b** shows representative cytokine array blots for each treatment.

H3-Modifications	*CAT 0.1%*			*CAT 0.2%*		
	**% of Control**	**SEM**	***p***	**% of Control**	**SEM**	***p***
Control	100	0		100	0	
H3K4me1	84.465	7.716	0.182	82.780	2.954	***0.004***
H3K4me2	97.308	1.966	0.304	89.369	4.559	0.080
H3K4me3	98.972	21.322	0.966	87.248	3.671	***0.025***
H3K9me1	103.973	4.417	0.463	98.647	9.821	0.897
H3K9me2	108.128	22.903	0.757	90.606	7.570	0.282
H3K9me3	99.416	10.702	0.961	85.968	5.170	***0.053***
H3K27me1	90.906	33.652	0.812	87.044	9.995	0.265
H3K27me2	101.486	0.518	0.103	83.539	6.305	***0.059***
H3K27me3	94.413	5.820	0.438	85.389	7.577	0.126
H3K36me1	98.275	8.165	0.852	91.826	4.629	0.152
H3K36me2	93.157	9.098	0.530	51.836	26.010	0.138
H3K36me3	92.571	9.807	0.528	78.633	7.170	***0.041***
H3K79me1	116.825	5.532	0.093	105.585	23.237	0.822
H3K79me2	114.195	9.359	0.269	105.411	11.249	0.656
H3K79me3	101.077	11.116	0.932	94.923	10.792	0.663
H3K9ac	96.606	3.994	0.485	81.099	8.896	0.101
H3K14ac	122.246	27.727	0.507	100.457	5.704	0.940
H3K18ac	104.309	6.956	0.599	83.617	3.803	***0.013***
H3K56ac	108.913	20.677	0.708	89.307	14.273	0.495
H3ser10P	100.256	20.977	0.991	91.570	3.400	0.068
H3ser28P	110.505	7.472	0.295	92.084	5.166	0.200

Supplementary Table T1: Representative table displaying modified H3 patterns after chronic alcohol exposure of human MDDCs *in vitro*. Table shows mean % of control, SEM and p value of each modification after 0.1 and 0.2% EtOH treatments. Modification experiments were performed twice for 0.1% EtOH and three times for 0.2% EtOH treated cells.

H4-Modifications	*CAT 0.2%*		
	% of Control	SEM	*p*
Control	100.000		
H4K5ac	15.439	28.305	**0.0113**
H4K8ac	33.691	12.891	**0.0002**
H4K12ac	401.642	123.397	**0.0309**
H4K16ac	15.988	66.118	0.2279
H4K20m1	96.476	20.220	0.8646
H4K20m2	53.625	10.554	**0.0009**
H4K20m3	59.632	15.015	**0.0197**
H4R3m2a	19.314	40.391	0.0689
H4R3m2	33.451	10.271	**0.0000**
H4ser1P	49.357	19.858	**0.0255**

Supplementary Table T2: Representative table displaying modified H4 patterns after chronic alcohol exposure of human MDDCs *in vitro*. Table shows histone modifications presented as a percentage over untreated control. The table shows mean % of control, SEM and p value of each modification compared to control. The H4 modification experiment was carried out 7 times.

CHRONIC

	Measured m/z	*Theoretical [M+H]⁺*	*error (ppm)*	*Sequence*	*Range*
Control	1180.62162	1180.62083	0.67	ISGLIYEETR	47 - 56
	989.57823	989.57784	0.39	VFLENVIR	61 - 68
	714.34609	714.34572	0.52	TLYGFGG	97 - 103
	515.34152	515.34124	0.54	LARR	38 - 41
Treated	1180.62198	1180.62083	0.97	ISGLIYEETR	47 - 56
	989.57842	989.57784	0.59	VFLENVIR	61 - 68
	714.34622	714.34572	0.7	TLYGFGG	97 - 103
	515.34157	515.34124	0.64	LARR	38 - 41

ACUTE

	Measured m/z	*Theoretical [M+H]⁺*	*error (ppm)*	*Sequence*	*Range*
Control	1180.62161	1180.62083	0.66	ISGLIYEETR	47 - 56
	989.57775	989.57784	-0.09	VFLENVIR	61 - 68
	714.34622	714.34572	0.70	TLYGFGG	97 - 103
	515.34117	515.34124	-0.14	LARR	38 - 41
Treated	1180.62107	1180.62083	0.20	ISGLIYEETR	47 - 56
	989.57822	989.57784	0.38	VFLENVIR	61 - 68
	714.34589	714.34572	0.24	TLYGFGG	97 - 103
	515.34118	515.34124	-0.12	LARR	38 - 41

Supplementary Table T3: Non-acetylated tryptic peptides detected by MALDI-FT-ICR MS for untreated control and MDDCs treated chronically or acutely with 0.2% EtOH. Notice that peptide sequences were detected with sub-ppm mass accuracy.
